# Phytophthora Disrupts Plant Immunity by Manipulating Nitric Oxide Homeostasis Through GSNOR Inhibition

**DOI:** 10.1002/advs.202503633

**Published:** 2025-06-20

**Authors:** Tingting Li, Jing Kang, Haizhu Zhang, Lina Wang, Minghui Lu, Lin Cai, Jianming Li, Matthieu H. A. J. Joosten, Yu Du

**Affiliations:** ^1^ State Key Laboratory for Crop Stress Resistance and High‐Efficiency Production College of Horticulture Northwest A&F University Yangling Shaanxi 712100 China; ^2^ College of Tobacco Science of Guizhou University/Key laboratory of Plant Resource Conservation and Germplasm Innovation in Mountainous Region (Ministry of Education)/Guizhou Key Lab of Agro‐Bioengineering Guiyang 550000 China; ^3^ Laboratory of Phytopathology Wageningen University Droevendaalsesteeg 1 Wageningen 6708 PB the Netherlands

**Keywords:** effector, GSNOR, phytophthora, plant defense, S‐nitrosylation

## Abstract

Nitric oxide (NO), a pivotal redox signaling molecule, coordinates plant development and immune responses through *S*‐nitrosylation‐mediated protein modification. While NO‐dependent *S*‐nitrosylation fine‐tunes immune responses, whether pathogens hijack this process to subvert plant immunity remains unclear. Here it is shown that *S*‐nitrosoglutathione reductase (GSNOR), which maintains NO homeostasis by degrading *S*‐nitrosoglutathione (GSNO), positively regulates tomato resistance to *Phytophthora capsici*. Active‐site mutations in GSNOR abolished its function in plant defense. Remarkably, GSNOR is manipulated by PcRD18, which is an RxLR effector of *P. capsici* that is involved in virulence of this oomycete pathogen. PcRD18 elevates the cellular NO content and *S*‐nitrosylation levels by dually inhibiting GSNOR activity and promoting its autophagy‐mediated degradation via enhanced ATG8c interaction. Structure analysis reveals critical PcRD18‐GSNOR interaction interfaces and mutations in these sites of PcRD18 abolish its ability to interact with GSNOR, thereby blocking the effector's ability to elevate NO levels, suppress the reactive oxygen species (ROS) burst, and enhance virulence. GSNOR mutations disrupting PcRD18 binding produced a mutant form of GSNOR enhancing *Phytophthora* resistance. These findings unveil a pathogen strategy to subvert NO homeostasis through effector‐mediated hijacking of GSNOR and suggest that engineering the host‐pathogen interface to disrupt the interaction between GSNOR and PcRD18 will enhance crop disease resistance.

## Graphical abstract

*Phytophthora capsici* effector PcRD18 targets tomato GSNOR to disrupt NO homeostasis, promoting *S*‐nitrosylation of immune proteins like RBOHD. This suppresses ROS bursts and promotes pathogen infection. Mutation of the PcRD18‐binding sites of GSNOR avoids pathogen manipulation and offers a strategy to enhance crop resistance.

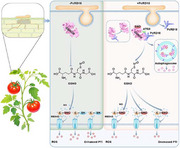

## 1 Introduction

Nitric oxide (NO), which is a pivotal signaling molecule, plays a crucial role in regulating development and stress responses in all living organisms^[^ ^1^, ^2^, ^3^ ^]^ NO primarily functions through *S*‐nitrosylation, which is a redox‐based posttranslational modification (PTM). During the *S*‐nitrosylation modification, an NO group is covalently attached to a thiol of a cysteine residue, forming an *S*‐nitrosothiol.^[^ ^2^ ^]^ *S‐*nitrosylation levels in cells are determined by the cytoplasmic NO concentration.

Like other PTMs, *S‐*nitrosylation modulates protein activity in numerous ways, including by altering their enzymatic activity, stability, and protein‐protein interactions.^[^ ^3^, ^4^ ^]^ The broad significance of this modification is evident across kingdoms. In mammals, *S*‐nitrosylation is implicated in diverse pathologies, including cancer, Parkinson's disease, and cardiovascular and neurodegenerative diseases.^[^ ^5^ ^]^ In plants, *S*‐nitrosylation governs a spectrum of processes spanning growth, development, and adaptation to stress.^[^ ^3^, ^6^ ^]^ For example, NO‐induced *S*‐nitrosylation of the stigma FERONIA receptor‐like kinase leads to reactive oxygen species (ROS) reduction and pollen growth, thereby regulating intra‐ and interspecific barriers in *Brassicaceae* crops.^[^ ^7^ ^]^ Alterations in *S*‐nitrosylation levels also influence root development, hypocotyl growth, fertility, and nodule development.^[^ ^6^, ^8^, ^9^, ^10^ ^]^ Beyond a role in development, *S*‐nitrosylation enhances tolerance to abiotic stresses, such as salinity and saline‐alkali conditions.^[^ ^11^, ^12^, ^13^ ^]^ Moreover, emerging studies have demonstrated that *S*‐nitrosylation plays a pivotal role in plant immunity regulation.^[^ ^14^ ^]^ Plants deploy a sophisticated immune system to combat pathogens. The first layer of plant immunity, referred to as pathogen‐associated molecular pattern (PAMP)‐triggered immunity (PTI), is activated when membrane‐localized pattern recognition receptors (PRRs) detect conserved PAMPs. This results in a burst of ROS, mitogen‐activated protein kinase (MAPK) signaling, and defense gene expression. Specialized pathogens, in turn, secrete effector proteins to suppress PTI, thereby promoting infection. As a countermeasure, plants have evolved effector‐triggered immunity (ETI), wherein intracellular immune receptors recognize these pathogen effectors, often triggering a more robust and rapid immune response.^[^ ^15^ ^]^ The precise coordination of immune responses relies on redox and hormonal signaling regulation, with emerging studies highlighting *S*‐nitrosylation as an additional vital regulatory mechanism in this process. Particularly, the activity of some key regulators involved in ROS synthesis, ROS scavenging, and SA signaling is regulated by modification through *S‐*nitrosylation.^[^ ^3^ ^]^ For example, *S‐*nitrosylation of the NADPH oxidase RESPIRATORY BURST OXIDASE HOMOLOG D (RBOHD) inhibits its enzymatic activity, thereby reducing the biosynthesis of ROS and inhibition of the hypersensitive response of plants.^[^ ^16^ ^]^ *S‐*nitrosylation of ASCORBATE PEROXIDASE (APX), which is a key enzyme involved in the scavenging of hydrogen peroxide, promotes its catalase activity, thereby lowering ROS levels and inhibiting the immune response of plants.^[^ ^17^ ^]^ Recent studies further demonstrate that *S*‐nitrosylation of tomato CAFFEIC ACID *O*‐METHYLTRANSFERASE 2 (SlCOMT2) stabilizes the enzyme to boost melatonin synthesis, which suppresses infection by the necrotrophic pathogen *Botrytis cinerea* by scavenging ROS and thereby inhibiting cell death.^[^ ^18^ ^]^ The SA binding activity of Arabidopsis SALICYLIC ACID BINDING PROTEIN 3 (AtSABP3) is suppressed after being *S‐*nitrosylated and hence leads to attenuated levels of SA‐related plant immunity.^[^ ^19^ ^]^ Moreover, *S‐*nitrosylation of the main regulatory factor NON EXPRESSOR OF *PR* GENES 1 (NPR1) of the SA signaling pathway promotes the production of NPR1 polymers, thereby inhibiting the expression of downstream *PATHOGENESIS‐RELATED* (*PR*) genes.^[^ ^20^ ^]^

*S‐*nitrosoglutathione reductase (GSNOR) belongs to the class III alcohol dehydrogenase (ADH3) family and catalyzes the reduction of *S‐*nitrosoglutathione (GSNO), which is the major cellular NO donor, to regulate the overall *S‐*nitrosylation level.^[^ ^21^ ^]^ The low levels of GSNOR will lead to an increased NO content and GSNO levels, as GSNO will not be reduced to glutathione disulfide and ammonia.^[^ ^21^ ^]^ Multiple studies have consistently demonstrated that NO plays a pivotal role in plant disease resistance, as well as in pathogen virulence. Therefore, GSNOR, as a crucial regulator of NO homeostasis and *S*‐nitrosylation levels, significantly impacts the complex interplay between plants and pathogens.^[^ ^3^, ^6^, ^22^ ^]^ However, it remains unknown whether pathogens deploy effectors specifically to target GSNOR, thereby disrupting NO homeostasis and *S*‐nitrosylation modification, as a strategy to suppress plant immunity.

Oomycetes, also referred to as water molds, are amongst the most damaging pathogens in agriculture. The genus *Phytophthora* contains more than 100 pathogens, including the most devastating pathogens on potato (*P. infestans*), soybean (*P. sojae*) and multiple important vegetable crops (*P. capsici*).^[^ ^23^, ^24^ ^]^ To infect the host, *Phytophthora* pathogens secrete a plethora of effectors into the apoplast and cytoplasm of the cells of the host, to suppress plant immunity. *Phytophthora* cytoplasmic effectors mainly include RxLR effectors, which is the most studied family of effectors and they are named after the Arginine‐any amino acid (x)‐Leucine‐Arginine consensus sequence, and CRINKLER effectors that are known for causing leaf crinkling and cell death.^[^ ^25^ ^]^ RxLR effectors are secreted from haustoria of oomycetes, and recent studies have demonstrated that RxLR effectors are internalized into plant cells through clathrin‐mediated endocytosis.^[^ ^26^, ^27^ ^]^ The genome of oomycetes is relatively simple and their effector proteins have clear sequence characteristics, thus providing a definite library of effector proteins. These features make oomycetes an excellent model for studying the biology of intracellular effector proteins. Notably, *P. capsici* demonstrates remarkable host plasticity, infecting over 26 different plant families, including *Solanaceae* (e.g., pepper and tomato), *Brassicaceae* (e.g., Arabidopsis), *Cucurbitaceae*, and *Leguminosae*.^[^ ^28^ ^]^ This broad host range, combined with the genetic tractability of model hosts like tomato and Arabidopsis,^[^ ^29^ ^]^ makes *P. capsici* an ideal oomycete pathogen for studying conserved effector‐target interactions across diverse plant lineages. In recent years, some biological functions of RxLR effectors from *P. infestans* and *P. sojae* have been revealed. However, from the 640 RxLR effectors of *P. capsici*, the biological function of only a dozen has been reported.^[^ ^30^, ^31^ ^]^ For example, several RxLR effectors from *P. capsici* have been found to disrupt the functioning of crucial immune regulators of host plants, including NPR1, receptor‐like cytoplasmic kinase (RLCK)‐VII proteins, ENHANCED DISEASE SUSCEPTIBILITY 1 (EDS1), the peptidyl‐prolyl isomerase FKBP15–2, and BINDING PARTNER OF ACD11‐1 (BPA1) that has been reported to play a negative role in the ROS‐mediated defense responses to *P. capsici* in Arabidopsis.^[^ ^32^, ^33^, ^34^, ^35^, ^36^ ^]^

In this study, we describe that the RxLR effector PcRD18 enhances the susceptibility of host plants to *P. capsici* by manipulating host GSNOR, to increase cellular NO and the overall *S‐*nitrosylation levels in the host cells with the aim to suppress plant immunity. Our results uncover a novel strategy whereby a plant pathogen suppresses immunity by disrupting the NO homeostasis and increasing host protein *S‐*nitrosylation.

## 2 Results

### 2.1 PcRD18 Promotes Plant Susceptibility During Phytophthora Infection

To identify effectors that are able to interfere with plant innate immunity, we collected a number of *P. capsici* RxLR effectors and performed inoculation assays using the *Nicotiana benthamiana* transient expression system to screen for RxLRs that enhance pathogen colonization. Among the screened effectors, the RxLR effector PcRD18 (*P. capsici* RxLR‐DEER‐18) drew our attention, as it was one of the RxLRs that markedly enhanced the susceptibility of *N. benthamiana* to *P. capsici* (Figure S1A, Supporting Information). Furthermore, inoculation tests showed that expression of GFP‐PcRD18 also promotes *P. capsici* infection in pepper (Figure S1B, Supporting Information). The GFP‐PcRD18 fusion protein was clearly detected by western blotting (Figure S2A, Supporting Information).

**Figure 1:**
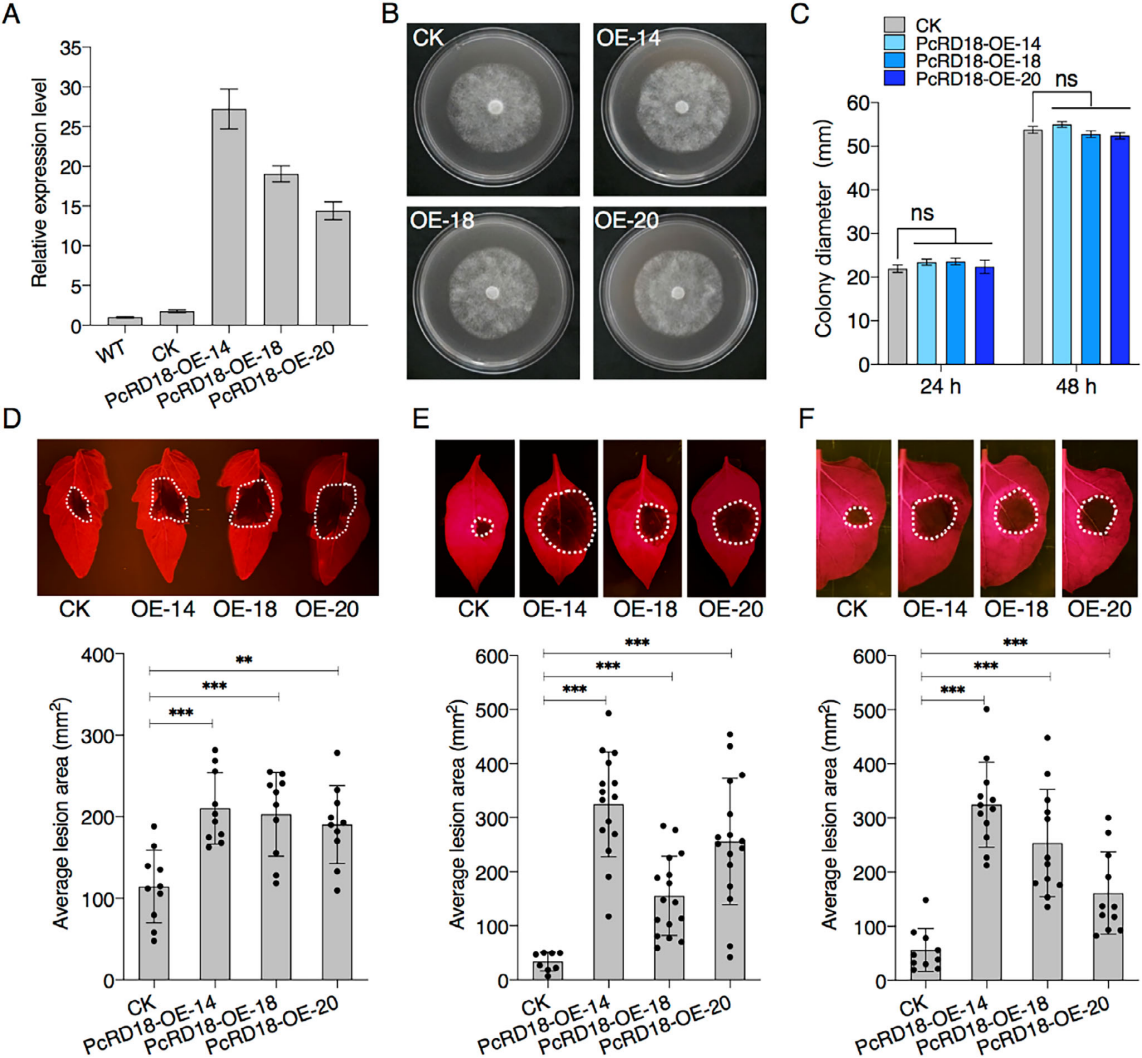
PcRD18 promotes *Phytophthora* virulence. A) Relative expression levels of *PcRD18* in three independent *PcRD18*‐overexpression lines (PcRD18‐OE), the wild type (*P. capsici* BYA5 strain) and the control line (CK), in which no DNA was integrated during the transformation process. Total RNA was isolated from the mycelium of *P. capsici* grown in liquid culture and RT‐qPCR was performed to determine the transcript levels of *PcRD18* in each sample. *PcActin* was used for normalization. Data are presented as mean ± SD (*n* = 3). B) Colonies of CK and PcRD18‐OE lines were cultured on V8 medium at 25 °C for 2 days and no difference in overall growth was observed. C) Statistical analysis of the colony diameters of the cultures shown in (B) at 24 and 48 h after plating. Statistical analysis was performed with two‐sided *t*‐tests (*n* = 6; ns, not significant). PcRD18 overexpressing strains of *P. capsici* exhibit enhanced virulence on tomato D), pepper E) and *N. benthamiana* F). Detached leaves were inoculated with *P. capsici* and photographed at 1.5 to 2 days after inoculation. Bar graphs indicate the average lesion areas. Statistical analysis was performed with two‐sided *t*‐tests (mean ± SD; n≥8; ^**^, *P* < 0.01; ^***^, *P* < 0.001). White dashed lines indicate the lesion areas. Each experiment was performed more than two times, with similar results.

To confirm the effect of PcRD18 on plant susceptibility to *P. capsici*, we generated stable *GFP‐PcRD18*‐transgenic tomato (*Solanum lycopersicum*, *Sl*) and Arabidopsis plants via *Agrobacterium*‐mediated transformation. By immunoblot assays, we verified the proper over‐expression (OE) of *GFP‐PcRD18* in the transgenic tomato plants, referred to as PcRD18‐SlOE (Figure S2B, Supporting Information), and in three Arabidopsis transgenic lines, indicated as PcRD18‐L1, ‐L2 and ‐L3 (Figure S2C, Supporting Information). Under standard growth conditions, PcRD18‐SlOE plants exhibit subtle growth retardation, which becomes evident from the six‐week‐old stage onward (Figure S2D, Supporting Information), whereas *PcRD18*‐transgenic Arabidopsis lines show no discernible phenotypic alterations (Figure S2E, Supporting Information). Detached leaves of 8‐week‐old tomato and 4‐week‐old Arabidopsis plants were used for inoculation tests. After inoculation with *P. capsici*, the lesions that developed on PcRD18‐SlOE tomato leaves were larger than those on the control wild type Ailsa Craig (AC) plants (Figure S1C, Supporting Information). Consistently, the PcRD18‐L1/L2/L3 Arabidopsis leaves were also more susceptible to *P. capsici*, when compared with the wild type Col‐0 control (Figure S1D, Supporting Information), suggesting that PcRD18 plays a role in virulence of the pathogen. To further investigate the function of PcRD18 in virulence of *P. capsici*, we overexpressed the *PcRD18* gene in the *P. capsici* strain BYA5. After confirmation via reverse transcription‐quantitative PCR (RT‐qPCR), we successfully obtained three independent *PcRD18* overexpression lines, designated PcRD18‐OE‐14, ‐18, and ‐20 (**Figure** **1**A). Additionally, a line that was taken along throughout the transformation procedure, but without transgene insertion was used as a control (CK). All three PcRD18‐OE lines showed similar morphology and mycelial growth when compared with the CK line (Figure 1B,C), whereas inoculation assays showed that PcRD18‐OE lines caused much larger disease lesions in tomato (Figure 1D), pepper (Figure 1E) and *N. benthamiana* (Figure 1F), when compared with the CK controls. Taken together, these results indicate that PcRD18 contributes to *P. capsici* virulence in several host plants.

### 2.2 PcRD18 Suppresses Plant Immune Responses

**Figure 2:**
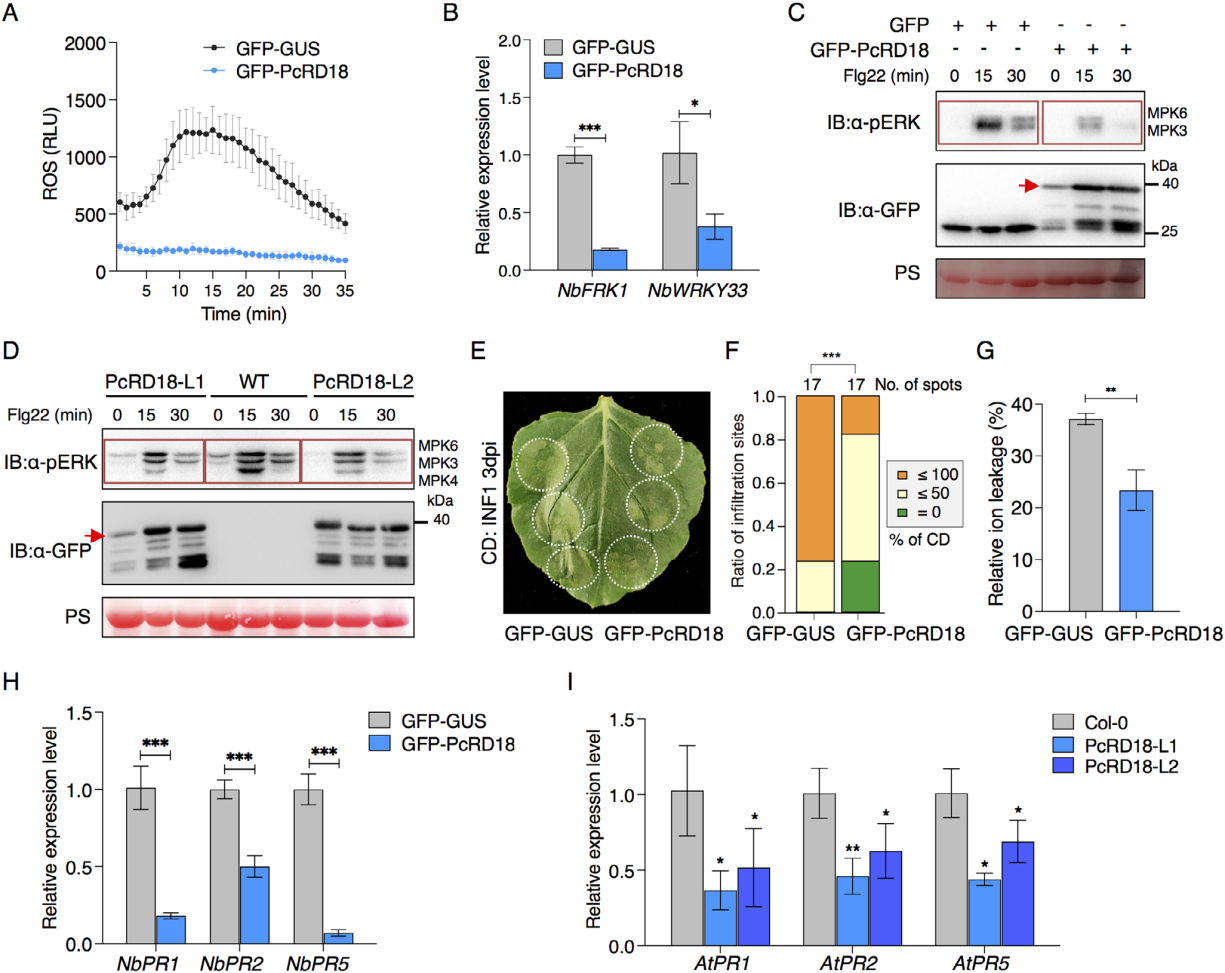
PcRD18 suppresses plant immunity. A) PcRD18 inhibits the flg22‐induced ROS burst in *N. benthamiana*. Leaf discs transiently expressing GFP‐PcRD18 or GFP‐GUS via agroinfiltration were used to measure the ROS burst at 0–35 min after treatment with 10 µM flg22. Data are presented as mean ± SD (n = 4). B) PcRD18 suppresses *NbFRK1* and *NbWRKY33* expression in *N. benthamiana*, as indicated by RT‐qPCR. Total RNA was isolated from *N. benthamiana* leaves expressing GFP‐PcRD18 or GFP‐GUS. Asterisks indicate significant differences (mean ± SD; *n* = 3; two‐sided *t*‐tests; ^*^, *P* < 0.05; ^***^, *P* < 0.001). Transient expression of PcRD18 in *N. benthamiana* C) or stable expression of PcRD18 in Arabidopsis D) suppresses flg22‐triggered MAPK activation. Total proteins were extracted from *N. benthamiana* leaves expressing GFP‐PcRD18 (GFP‐GUS as control) and two independent transgenic lines (wild‐type Col‐0 as control) at 0, 15, and 30 min post‐treatment with 10 µm flg22. MAPK activation was detected via immunoblotting using an anti‐pERK antibody. Ponceau S (PS) staining confirmed total protein loading, with the rubisco band serving as a reference. Red arrows mark the GFP‐PcRD18 protein. E–G) PcRD18 suppresses INF1‐induced plant cell death. INF1 was co‐expressed with GFP‐PcRD18 (or control GFP‐GUS) in *N. benthamiana*, and representative images were captured at 3 days post‐infiltration (dpi). White dashed circles indicate the infiltration areas. Both the cell death level analysis (one‐sided Wilcoxon rank‐sum test; ^***^, *P* < 0.001) in F) and the relative ion leakage assay (mean ± SD; one‐sided *t*‐test; **, *P* < 0.01) in G) indicate suppression of cell death by PcRD18. Cell death (CD) levels were divided over three categories in F), based on the percentage of necrosis in infiltrated areas (0%, 0%–50%, and 50%–100%). Transient expression of *PcRD18* in *N. benthamiana* H) or stable expression of *PcRD18* in Arabidopsis **(I)** inhibits the expression of SA‐related *PR* genes. Total RNA was extracted from leaves of *N. benthamiana* expressing *GFP‐PcRD18* or *GFP‐GUS* at 2 dpi in (H) and from leaves of *GFP‐PcRD18*‐transgenic Arabidopsis lines or Col‐0 in I). *NbActin* and *AtActin* served as reference genes for RT‐qPCR normalization. Two‐sided *t*‐tests were used in statistical analyses (mean ± SD; *n* = 3; ^*^, *P* < 0.05; ^**^, *P* < 0.01; ^***^, *P* < 0.001).

Considering that PcRD18 facilitates *Phytophthora* infection, we wondered whether PcRD18 interferes with plant innate immunity, including the ROS burst, defense‐related gene expression and MAPK activation. To explore this possibility, we first examined the flg22‐induced ROS burst in leaves of *N. benthamiana* transiently expressing GFP‐PcRD18 or GFP‐GUS. The results indicated that expression of PcRD18 nearly abolished the flg22‐induced ROS burst, whereas GFP‐GUS did not affect this response (**Figure** **2**A). Next, we determined whether PcRD18 affects the expression levels of the PTI marker genes FLAGELLIN 22‐INDUCED RECEPTOR‐LIKE KINASE 1 (*FRK1*) and the gene encoding transcription factor WRKY33. RT‐qPCR data showed that transient expression of GFP‐PcRD18 in leaves of *N. benthamiana* significantly decreased the expression of both *FRK1* and *WRKY33* (Figure 2B). Besides, activation of the MAPK cascade in *N. benthamiana* was determined in GFP‐PcRD18‐ and GFP‐GUS‐expressing leaves upon flg22 treatment. Immunoblot assays indicated that flg22‐induced MAPK activation was largely suppressed by PcRD18 (Figure 2C). This observation was further confirmed in Arabidopsis *PcRD18*‐transgenic lines (Figure 2D). Moreover, we performed cell death assays in *N. benthamiana* to test the effect of PcRD18 on PAMP‐induced cell death. *GFP‐PcRD18*, or the control *GFP‐GUS*, was co‐infiltrated with *INF1*, which encodes a well‐known oomycete PAMP elicitor.^[^ ^37^ ^]^ At 3‐days post infiltration (dpi), INF1‐triggered cell death occurred in areas expressing GFP‐GUS, but was much reduced in the areas expressing GFP‐PcRD18 (Figure 2E). Determination of the level of cell death (Figure 2F) and ion leakage assays (Figure 2G), showed that cell death was suppressed significantly by GFP‐PcRD18. It is known that the SA signaling pathway plays vital roles in plant immunity and that activation of the SA signaling pathway correlates with ROS and cell death. We therefore also examined the expression levels of the SA marker genes *PATHOGENESIS‐RELATED 1* (*PR1*), *PR2*, and *PR5* in PcRD18‐transiently‐ and stably‐expressing leaves. RT‐qPCR results showed that the expression of these *PR* genes was repressed significantly in both transiently *GFP‐PcRD18*‐expressing *N. benthamiana* leaves (Figure 2H) and in stable *GFP‐PcRD18*‐transgenic Arabidopsis lines (Figure 2I). These results indicate that PcRD18 suppresses plant immunity.

### 2.3 PcRD18 Interacts with SlGSNOR

To identify PcRD18 host target proteins, we transiently expressed GFP‐PcRD18 in leaves of *N. benthamiana* and performed immunoprecipitation (IP) with anti‐GFP Trap beads to obtain co‐purifying PcRD18‐interacting host proteins. Subsequent tryptic digestion of the immunoprecipitate, followed by liquid chromatography–mass spectrometry/mass spectrometry (LC–MS/MS) of the peptides was carried out to identify the candidate interacting proteins of PcRD18. We eventually identified 345 candidate interacting proteins after excluding the proteins that showed non‐specific binding to the GFP control (Table S1, Supporting Information). One of the candidates, GSNOR that was present among the top 30 of candidate‐interacting proteins, was selected for further studies, due to its described involvement in the ROS burst, cell death and SA‐related defense responses,^[^ ^3^, ^16^, ^20^, ^38^, ^39^ ^]^ which are all suppressed by PcRD18 (Figure 2).

**Figure 3:**
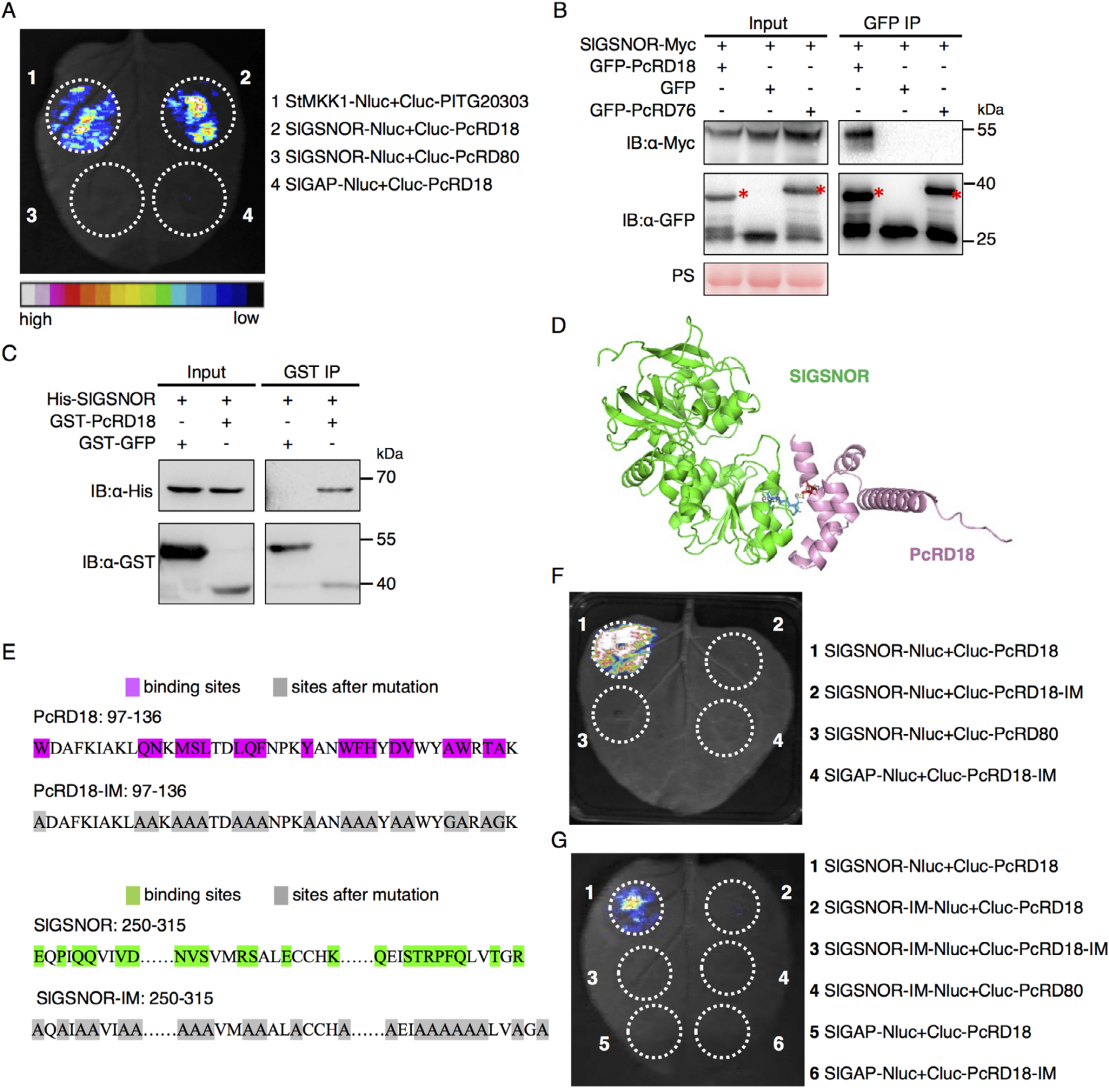
The interaction between PcRD18 and SlGSNOR. A) Interaction between PcRD18 and SlGSNOR as revealed by a firefly luciferase complementation imaging (LCI) assay. StMKK1‐Nluc and Cluc‐PITG20303 were co‐expressed as the positive control in *N. benthamiana* leaves (1, upper left circle). SlGSNOR‐Nluc and Cluc‐PcRD18 were co‐expressed to determine the interaction (2, upper right circle). SlGSNOR‐Nluc with Cluc‐PcRD80 (3, lower left circles) and SlGAPDH‐Nluc with Cluc‐PcRD18 (4, lower right circle) were co‐expressed as negative controls. The numbers indicate different infiltrations. Luminescence images were acquired with a CCD‐camera at 2 dpi. B) Co‐IP assay showing that PcRD18 associates with SlGSNOR *in planta*. Total proteins were isolated from *N. benthamiana* leaves transiently expressing the indicated constructs, at 2 days after agro‐infiltration. GFP‐Trap beads (GFP IP) were used for the immunoprecipitation. The red asterisks indicate GFP‐PcRD18 and the control effector GFP‐PcRD76. Total protein loading is indicated by the rubisco band from the Ponceau staining (PS). C) PcRD18 interacts with SlGSNOR in vitro. Proteins were produced in *Escherichia coli* and His‐SlGSNOR was incubated with GST‐PcRD18 or GST‐GFP in vitro for GST pull‐down assays. D) Modeling of the structure of the SlGSNOR‐PcRD18 complex, as predicted by AlphaFold using MMseqs2 (ColabFold). E) The interaction sites in PcRD18 and SlGSNOR were predicted by PyMOL. The interaction sites in PcRD18 are highlighted in purple, whereas the interaction sites in SlGSNOR are highlighted in green. The mutated sites are highlighted in gray. PcRD18‐IM and SlGSNOR‐IM indicate the mutants of PcRD18 or SlGSNOR, respectively. Mutating the predicted interacting sites in PcRD18 F) or in SlGSNOR G) abolished the interaction between SlGSNOR and PcRD18, as indicated by the LCI assay. Each experiment was repeated more than three times, with similar results.

A firefly luciferase complementation imaging (LCI) assay was subsequently performed to detect the *in planta* interaction between tomato GSNOR (SlGSNOR) and PcRD18. StMKK1‐Nluc and Cluc‐PITG20303 were co‐expressed as a positive control.^[^ ^40^ ^]^ The results indicate that co‐expression of SlGSNOR‐Nluc and Cluc‐PcRD18 in *N. benthamiana* activates luciferase activity (**Figure** **3**A). PcRD80, which is another RxLR effector from *P. capsici*, and tomato GLYCERALDEHYDE‐3‐PHOSPHATE DEHYDROGENASE (SlGAPDH), which is one of the candidates PcRD80‐interacting proteins identified by LC‐MS/MS were used as controls in the LCI assay. Co‐expression of SlGSNOR‐Nluc and Cluc‐PcRD80 or SlGAPHD‐Nluc and Cluc‐PcRD18 did not result in any luciferase activity (Figure 3A). To confirm the interaction, we co‐expressed SlGSNOR‐Myc with GFP‐PcRD18, GFP only or GFP‐PcRD76, which is yet another RxLR effector from *P. capsici* that is used as a control, in *N. benthamiana* and performed co‐immunoprecipitation (co‐IP) assays. The results show that SlGSNOR‐Myc co‐immunoprecipitates with GFP‐PcRD18, but not with GFP only or with GFP‐PcRD76 (Figure 3B). Consistently, pull‐down assays confirmed the interaction between His‐SlGSNOR and GST‐PcRD18 in vitro (Figure 3C). Moreover, mCherry‐PcRD18 and GFP‐SlGSNOR were co‐expressed in *N. benthamiana* leaves to examine whether PcRD18 co‐localizes with SlGSNOR. The confocal images reveal overlapping fluorescence of GFP‐SlGSNOR and mCherry‐PcRD18 within both the nucleus and cytoplasm (Figure S3, Supporting Information), thereby confirming their co‐localization. Taken together, these results reveal that the RxLR effector PcRD18 interacts, and co‐localizes with SlGSNOR. To determine whether PcRD18 interacts with CaGSNOR, the pepper ortholog of SlGSNOR, we conducted co‐IP assays. Results demonstrated that Myc‐PcRD18, but not the control RxLR effector Myc‐PcRD324, was specifically co‐immunoprecipitated with GFP‐CaGSNOR when transiently co‐expressed in *N. benthamiana* (Figure S4, Supporting Information), suggesting that GSNOR represents a conserved virulence target of PcRD18 in diverse host plants of *P. capsici*.

To gain structural insight into how the effector PcRD18 interacts with SlGSNOR, we constructed a 3D model of the PcRD18‐SlGSNOR complex (Figure 3D), as predicted by ColabFold,^[^ ^41^ ^]^ which combines the fast homology search of MMseqs2 with AlphaFold2.^[^ ^42^ ^]^ The LigPlot prediction suggested potential hydrogen bonding between the residues Asp127 (PcRD18) and Arg279 (SlGSNOR) (Figure S5A, Supporting Information). However, subsequent mutagenesis analysis revealed that neither residue is essential for the interaction, as the PcRD18^D127A^ and SlGSNOR^R279A^ mutants retained their binding capacity in LCI assays (Figure S5B,C, Supporting Information). Next, the interacting sites in the PcRD18‐SlGSNOR complex were also predicted by PyMOL and these sites are shown highlighted in purple in PcRD18 and highlighted in green in SlGSNOR (Figure 3E). By mutating the amino acid residues present at the predicted interaction sites of PcRD18 into alanine or glycine as shown in Figure 3E, we obtained the PcRD18‐Interacting‐sites Mutant (PcRD18‐IM). Further LCI assays showed that the PcRD18‐IM now has lost the ability to interact with SlGSNOR (Figure 3F). Furthermore, the SlGSNOR‐Interacting‐sites Mutant (SlGSNOR‐IM) also has lost the ability to interact with PcRD18 in an LCI assay (Figure 3G). Both PcRD18‐IM and SlGSNOR‐IM were properly expressed, as determined by western blotting (Figure S6, Supporting Information). These results suggest that the predicted sites are indeed required for the interaction between PcRD18 and SlGSNOR.

### 2.4 GSNOR Positively Regulates Plant Resistance to *P. capsici*, and its Enzymatic Activity is Required for its Function in Immunity

**Figure 4:**
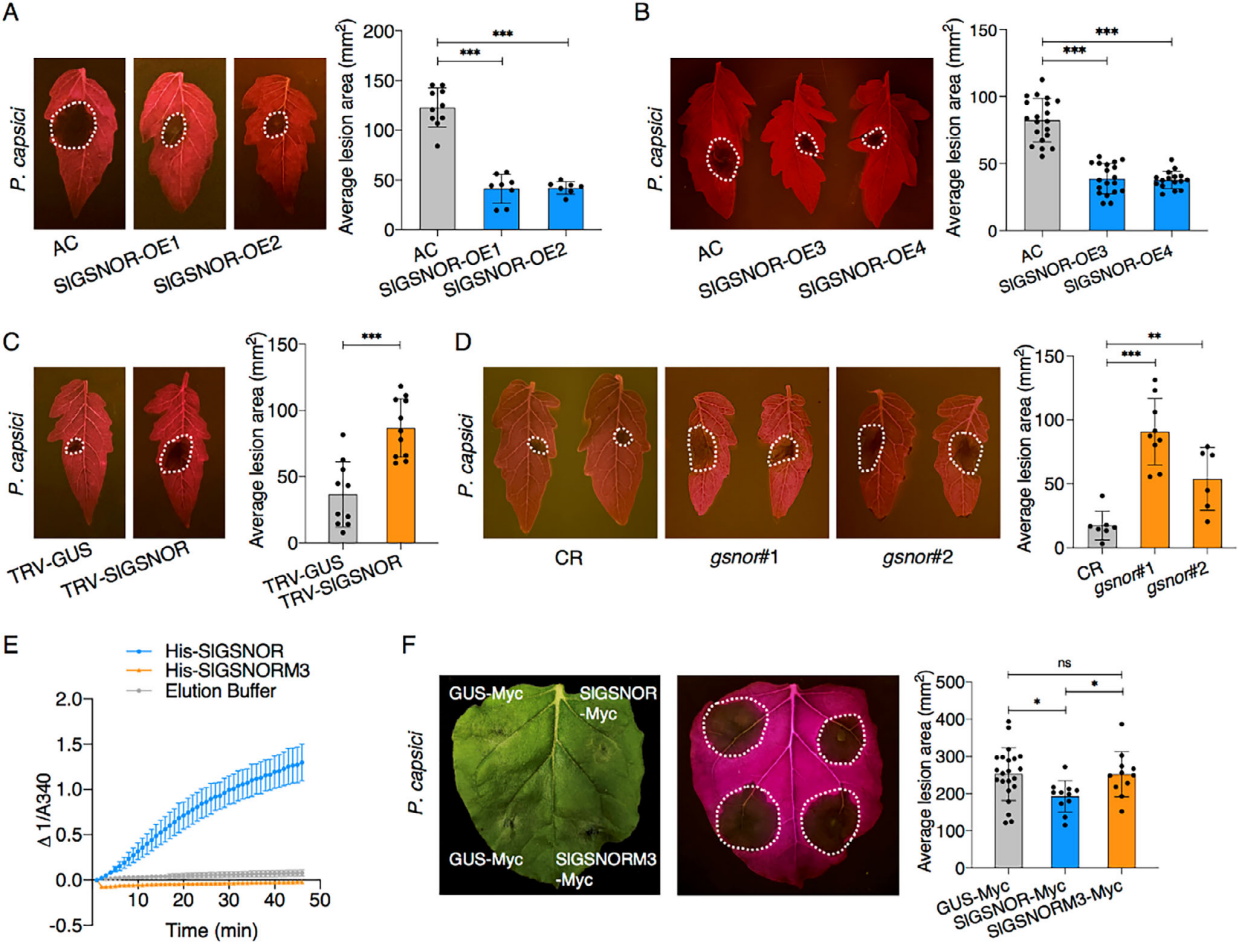
GSNOR positively regulates plant defense to *P. capsici*. *SlGSNOR* over‐expressing tomato lines SlGSNOR‐OE1/OE2 A) and SlGSNOR‐OE3/OE4 B) exhibit enhanced resistance to *P. capsici*, as compared to the wild‐type tomato cultivar Ailsa Craig (AC). SlGSNOR‐OE1 and SlGSNOR‐OE2 represent independent transgenic lines expressing GFP‐SlGSNOR. SlGSNOR‐OE3 and SlGSNOR‐OE4 are previously described overexpression lines.^[^ ^43^ ^]^ Leaves of 5‐8‐week‐old tomato plants were used for *P. capsici* inoculation. Silencing C) or knocking out D) of *SlGSNOR* in tomato increases *P. capsici* leaf colonization. Leaves from TRV‐*SlGSNOR*‐ and TRV‐*GUS‐*inoculated plants were inoculated with *P. capsici* in (C). Leaves of 5‐week‐old *SlGSNOR* knock‐out lines *gsnor*#1, #2 and the wild type CR (Condine Red) were inoculated with *P. capsici* in (D). Lesions were photographed (left panel) and their area was measured at 2 to 2.5 days after inoculation (dai).The bar graphs (right panel) show the lesion area analysis (mean ± SD; n≥6; two‐sided *t*‐tests; ^**^, *P* < 0.01; ^***^, *P* < 0.001) in (A–D). E) Mutations in the binding sites for catalytic Zn^2+^ abolish the enzymatic activity of SlGSNOR. SlGSNORM3 (SlGSNOR^C47A, H69A, E70A, C177A^) represents the mutant of the Zn^2+^ binding sites. His‐SlGSNOR and His‐SlGSNORM3 were expressed in vitro and used for GSNOR activity assays. The elution buffer used in the protein purification protocol was used as a negative control and GSNO was used as a substrate. The absorbance at 340 nm (A340) was determined to measure the decomposition of NADH in the enzyme reaction mix and the relative enzymatic activity is indicated by △1/A340. Data are presented as mean ± SD (*n* = 3). F) Enzymatic activity of SlGSNOR is required for its function in resistance to *P. capsici*. *N. benthamiana* leaves transiently expressing SlGSNOR‐Myc, SlGSNORM3‐Myc and GUS‐Myc were inoculated with *P. capsici* at 24 h after agro‐infiltration (left panel). The bar graph (right panel) shows the average lesion areas measured at 2 dai (mean ± SD; n>10; two‐sided *t*‐tests; ^*^, *P* < 0.05; ns, not significant). Dashed white lines in the leaves indicate the lesion areas.

To explore the role of GSNOR in tomato defense against *P. capsici*, we transformed *GFP*‐*SlGSNOR* of which the expression is driven by the cauliflower mosaic virus 35S promoter, into tomato via *Agrobacterium*‐mediated stable transformation and obtained two independent transgenic lines, SlGSNOR‐over expressor (OE) 1 and SlGSNOR‐OE2. Immunoblotting (Figure S7A, Supporting Information) and RT‐qPCR (Figure S7B, Supporting Information) confirmed the accumulation of the intact GFP‐SlGSNOR fusion protein and the increased transcription levels of *SlGSNOR* in the transgenic lines, when compared to the untransformed wild‐type tomato cultivar Ailsa Craig (AC). The stable‐transformed tomato plants showed no visible morphological differences when compared to AC (Figure S7C, Supporting Information). Detached leaves of SlGSNOR‐OE1, SlGSNOR‐OE2 and AC plants were inoculated with *P. capsici*, and statistical analysis showed that the lesions that developed on SlGSNOR‐OE leaves were smaller than those developing on the leaves of the wild type plants (**Figure** **4**A). Furthermore, two additional SlGSNOR‐overexpressing lines (SlGSNOR‐OE3 and SlGSNOR‐OE4) described in a previous study,^[^ ^43^ ^]^ exhibited similar resistance levels (Figure 4B). Moreover, we obtained *SlGSNOR‐*silenced tomato lines by virus‐induced gene silencing (VIGS) using recombinant tobacco rattle virus (TRV) and determined the silencing efficiency by RT‐qPCR (Figure S8A, Supporting Information). Growth of TRV‐*SlGSNOR*‐inoculated plants did not show any difference when compared with the control TRV‐*GUS‐*inoculated plants, except for spontaneous cell death occurring in some leaves of the TRV‐*SlGSNOR* plants (Figure S8B, Supporting Information). Upon performing *P. capsici* inoculation of the leaves of the TRV‐*SlGSNOR*‐ and the control TRV‐*GUS*‐inoculated plants, statistical analysis indicated that silencing of *SlGSNOR* significantly increased the lesions areas (Figure 4C). Consistently, the leaves from tomato lines *gsnor*#1 and *gsnor*#2, in which *SlGSNOR* has been knocked out,^[^ ^44^ ^]^ exhibited enlarged lesions in comparison to those from the wild‐type Condine Red (CR) (Figure 4D). To validate the conserved role of GSNOR across *P. capsici* hosts, we extended our analysis to pepper and tobacco. Transient overexpression of *CaGSNOR* significantly increased disease resistance of pepper leaves (Figure S9A, Supporting Information). Conversely, inhibition of CaGSNOR using N6022, a GSNOR‐specific inhibitor, increased symptom development, resulting in larger lesion areas than the 0.1% DMSO control (Figure S9B, Supporting Information). Parallel experiments in *N. benthamiana* demonstrated that transient expression of *SlGSNOR‐Myc* conferred enhanced resistance compared to the *GUS‐Myc* control (Figure S10A, Supporting Information). Consistent with the gain‐of‐function analyses, VIGS‐mediated silencing of *NbGSNOR* (Figure S11, Supporting Information) rendered plants more susceptible, exhibiting enlarged lesions compared to TRV‐*GUS* controls following *P. capsici* challenge (Figure S10B, Supporting Information), further supporting a positive regulatory role of NbGSNOR in plant defense. Together, these results demonstrate that GSNOR positively regulates defense against *P. capsici* in tomato, pepper, and tobacco.

GSNOR belongs to the ADH3 family and specifically catalyzes GSNO.^[^ ^21^, ^45^ ^]^ To explore whether the enzymatic activity of SlGSNOR is required for its function in plant immunity, we constructed the mutant SlGSNORM3 (SlGSNOR^C47A, H69A, E70A, C177A^), carrying amino acid residue changes that affect the binding site for catalytic Zn^2+^, according to the reported crystal structure of SlGSNOR.^[^ ^46^ ^]^ In vitro enzymatic activity assays confirmed that the mutations in SlGSNORM3 abolished the enzymatic activity of SlGSNOR (Figure 4E). When transiently expressed in *N. benthamiana*, SlGSNOR‐Myc decreases *P. capsici* colonization as compared with expression of GUS‐Myc, while SlGSNORM3‐Myc expression resulted in similar lesions as expression of GUS‐Myc and showed significantly larger lesions than upon expression of SlGSNOR‐Myc (Figure 4F), suggesting that SlGSNORM3‐Myc has lost the ability to enhance plant resistance. Both SlGSNOR‐Myc and SlGSNORM3‐Myc were correctly expressed as indicated by western blotting (Figure S12, Supporting Information). These results indicate that the enzymatic activity of SlGSNOR is required for its function in plant immunity.

### 2.5 SlGSNOR Positively Regulates Plant PTI Responses

**Figure 5:**
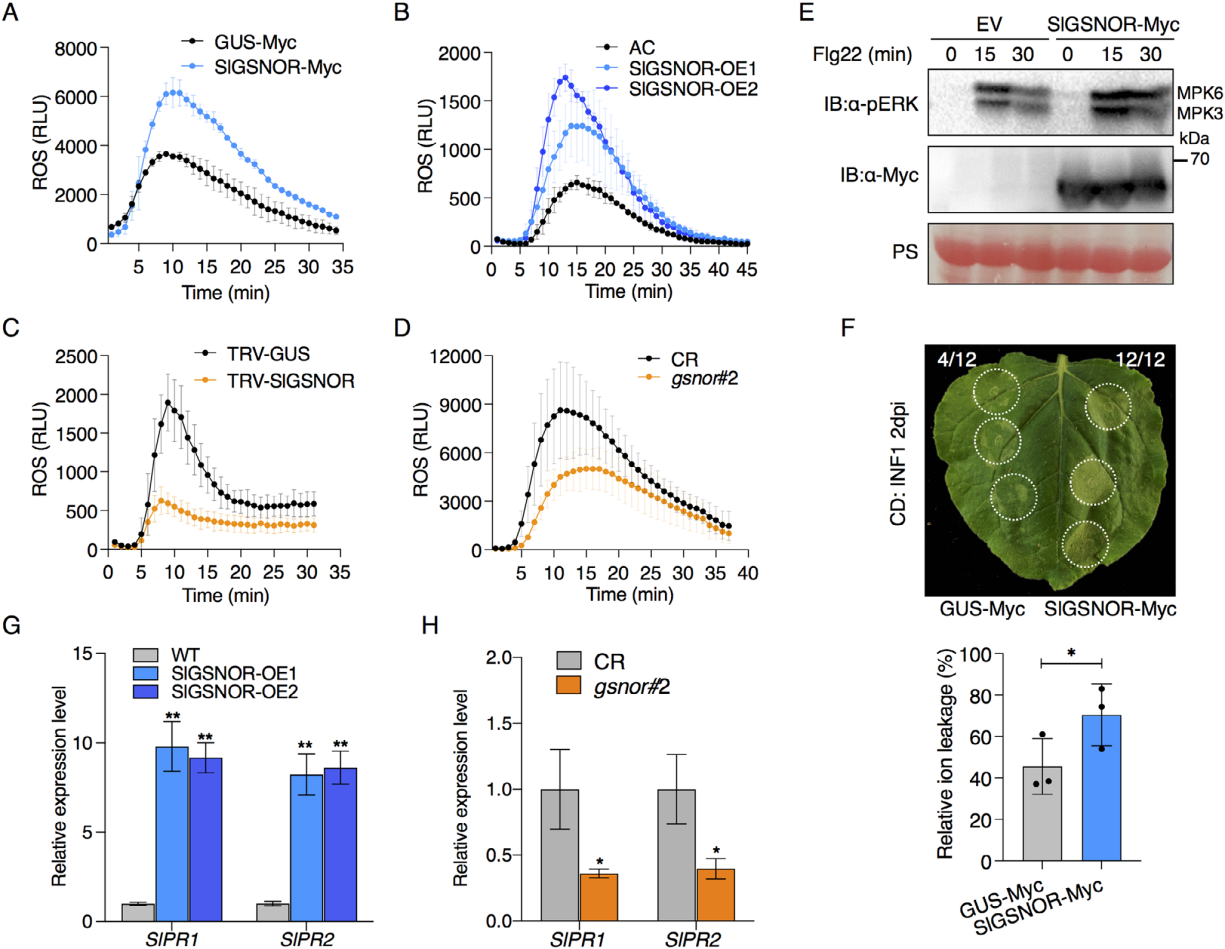
SlGSNOR positively regulates plant PTI responses. A) Transient expression of *SlGSNOR* in *N. benthamiana* increases the flg22‐induced ROS burst. *SlGSNOR‐Myc* and the control *GUS‐Myc* were transiently expressed in *N. benthamiana* leaves and after 2 days the ROS burst, shown in relative light units (RLU), was measured at 0–35 min after treatment with 10 µm flg22. Values represent the means ± standard deviation (SD) (*n* = 3). B) Overexpression of *SlGSNOR* in tomato increases the flg22‐induced ROS burst. Leaf discs from 8‐week‐old SlGSNOR overexpressing lines SlGSNOR‐OE1, and ‐OE2, and wild type AC (Ailsa Craig) tomato plants were used for ROS determination. Values represent the means ± SD (*n* = 3). Flg22‐induced ROS burst in *SlGSNOR*‐silenced C) and knock‐out D) transgenic tomato lines. Leaf discs from TRV‐*SlGSNOR*‐inoculated (with TRV‐*GUS*‐inoculated plants as a control), and knock‐out *gsnor* tomato plants (with wild type Condine Red (CR) used as a control), were used for ROS determination. Values represent the means ± SD (*n* = 4). E) Expression of SlGSNOR in *N. benthamiana* enhances MAPK activation, induced by flg22. Total proteins were extracted from *N. benthamiana* expressing *SlGSNOR‐Myc* and the empty vector (EV) control, after treatment with 10 µm flg22 for 0, 15, and 30 min. An anti‐pERK antibody was used to examine MAPK activation. Total protein loading is shown by Ponceau S (PS) staining, based on the intensity of the rubisco band. F) Expression of *SlGSNOR* in *N. benthamiana* increases INF1‐induced cell death (CD). *INF1* was co‐expressed with *SlGSNOR‐Myc* or the *GUS‐Myc* control, respectively. Cell death was photographed (upper panel) and quantified at 2 days post agro‐infiltration (dpi) by determining the relative ion leakage levels (lower panel). In the upper panel, the ratios indicated above the leaf indicate the number of infiltration sites for which cell death has occurred, versus the total number of infiltration sites. The bar graph in the lower panel shows the relative ion leakage (mean ± SD; *n* = 3; two‐sided *t*‐test; ^*^, *P* < 0.05). *PR* gene expression in the *SlGSNOR* overexpressing transgenic tomato lines G) and in the knock‐out line *gsnor* H). Total RNA was isolated from 8‐week‐old SlGSNOR overexpressing tomato lines SlGSNOR‐OE1, ‐OE2 and from the wild type AC in (G). Leaves from the 5‐week‐old *SlGSNOR* knock‐out line *gsnor* and the wild type CR plants were used for RNA extraction in H). Gene expression levels were determined by RT‐qPCR and normalized to the internal control gene *SlActin* (mean ± SD; *n* = 3; two‐sided *t*‐tests; ^*^, *P* < 0.05; ^**^, *P* < 0.01).

To confirm the molecular mechanism of SlGSNOR positively regulating disease resistance, we first determined the PTI responses that the effector PcRD18 suppresses, including the ROS burst, MAPK activation and PAMP‐induced cell death. When SlGSNOR‐Myc was transiently expressed in *N. benthamiana*, the ROS burst induced by flg22 was greatly elevated compared with leaves transiently expressing the GUS‐Myc control (**Figure** **5**A). To confirm this observation in tomato, we determined the flg22‐induced ROS burst in *SlGSNOR* overexpression (SlGSNOR‐OE), silenced (TRV‐*SlGSNOR*‐inoculated), and knock‐out (*gsnor*#2) plants. The results showed that overexpression of *SlGSNOR* in tomato increased the ROS burst (Figure 5B), while silencing (Figure 5C) or knocking out of *SlGSNOR* decreased the ROS burst (Figure 5D). Consistently, MAPK activation induced by flg22 at 15 and 30 min was enhanced upon expression of SlGSNOR in *N. benthamiana* (Figure 5E). Cell death triggered by the elicitor INF1 was increased upon co‐expression with SlGSNOR‐Myc (Figure 5F) and was decreased in leaves of TRV‐*NbGSNOR*‐inoculated plants (Figure S13, Supporting Information). In addition, we examined the expression of SA marker genes in SlGSNOR‐OE and *gsnor* lines. RT‐qPCR data showed that overexpression of *SlGSNOR* resulted in upregulated *PR1* and *PR2* expression (Figure 5G), whereas knocking out *SlGSNOR* in tomato caused downregulation of their expression (Figure 5H). These results indicate that SlGSNOR positively regulates the various PTI responses that we quantified.

### 2.6 PcRD18 Inhibits the Enzymatic Activity and Protein Stability of SlGSNOR

**Figure 6:**
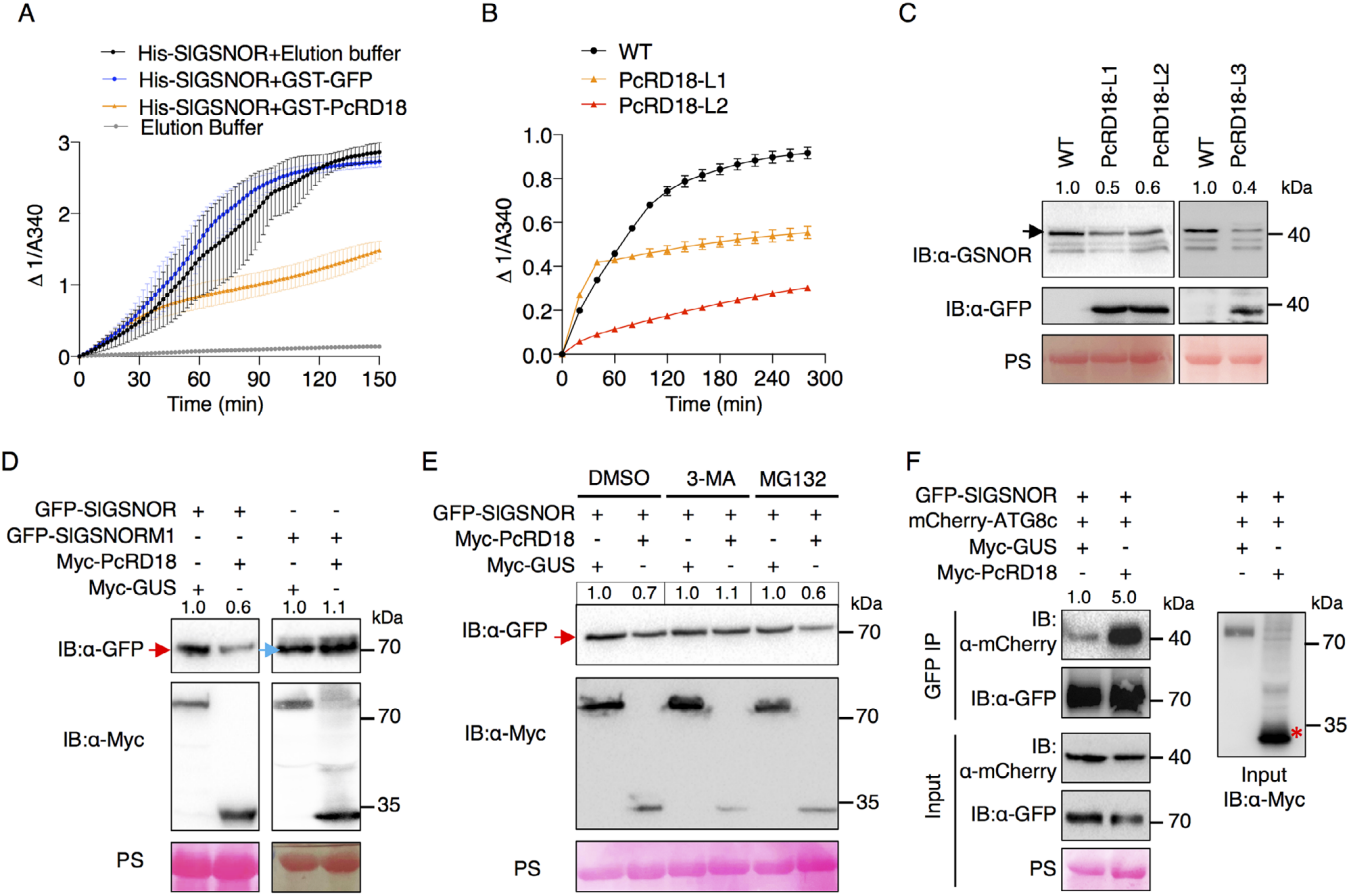
PcRD18 suppresses the enzymatic activity and stability of SlGSNOR. A) PcRD18 suppresses the enzymatic activity of SlGSNOR in vitro. The enzymatic activity of His‐SlGSNOR was measured in vitro upon incubation with GST‐PcRD18, GST‐GFP or elution buffer. The GFP‐PcRD18‐transgenic Arabidopsis lines exhibit a decreased GSNOR activity B) and lower GSNOR protein accumulation C), when compared with the wild type, Col‐0. Total proteins were extracted from 4‐week‐old Arabidopsis leaves. The relative enzyme activity is shown by the consumption of NADH, as indicated by △1/A340 in (A,B) and data are presented as mean ± SD (*n* = 3). PcRD18‐L1, ‐L2 and ‐L3 represent three independent *GFP‐PcRD18*‐transgenic Arabidopsis lines. Anti‐GSNOR antibody is used to detect GSNOR protein accumulation levels (black arrow). D) PcRD18 co‐expression reduces SlGSNOR accumulation but not its non‐nitrosylatable mutant SlGSNORM1. Total proteins were extracted from *N. benthamiana* leaves transiently co‐expressing GFP‐SlGSNOR or SlGSNORM1, either with Myc‐PcRD18 or with Myc‐GUS, at 48 h post infiltration (hpi). SlGSNORM1 represents the mutant SlGSNOR^C10/271/370S^. E) The destabilization of SlGSNOR by PcRD18 is dependent on the autophagy pathway. GFP‐SlGSNOR was co‐expressed either with Myc‐PcRD18 or with Myc‐GUS in *N. benthamiana*. MG132, 3‐methyladenine (3‐MA), or the 0.1% DMSO solvent as a negative control was infiltrated into the agro‐infiltrated areas at 36 hpi and total proteins were isolated from samples harvested at 48 hpi. + and – indicate transient expression of the protein in the leaves or not, respectively. The numbers above the bands in (C–E) indicate the relative intensity of the bands representing AtGSNOR (black arrow), GFP‐SlGSNOR (red arrows) or GFP‐SlGSNORM1 (blue arrow) proteins, normalized to the Rubisco level. F) PcRD18 enhances the interaction between SlGSNOR and ATG8c. GFP‐SlGSNOR and mCherry‐ATG8c were co‐expressed with either Myc‐PcRD18 or the control Myc‐GUS in *N. benthamiana*. Total proteins were extracted at 48 hpi and GFP‐Trap beads (GFP IP) were used in the immunoprecipitation assay. The number above the band indicates the ratio of mCherry‐ATG8c band intensities in samples without or with Myc‐PcRD18. The red asterisks indicate Myc‐PcRD18. Ponceau S staining (PS) of Rubisco serves as a protein loading control in (C–F).

To explore whether the RxLR effector PcRD18 of *P. capsici* targets SlGSNOR to suppress its enzymatic activity, we purified the His‐SlGSNOR protein and measured its catalytic activity toward GSNO, either in the presence of GST‐PcRD18 or the control GST‐GFP protein. The results show that GST‐PcRD18 significantly suppresses SlGSNOR enzymatic activity, as compared with the control GST‐GFP or the elution buffer (**Figure** **6**A). Furthermore, in vivo GSNOR enzyme activity assays consistently showed that *PcRD18*‐transgenic Arabidopsis lines PcRD18‐L1 and ‐L2 exhibit a decreased GSNOR activity when compared with the wild type Col‐0 (Figure 6B).

It is known that Arabidopsis GSNOR is degraded via selective autophagy upon its *S‐*nitrosylation.^[^ ^47^ ^]^ This feature led us to hypothesize that the protein accumulation of SlGSNOR also may be affected by PcRD18. We therefore determined the protein accumulation levels of GSNOR in PcRD18‐L1, ‐L2, and ‐L3 transgenic lines, using a specific anti‐GSNOR antibody. Western blot analysis revealed a significant reduction in GSNOR protein levels across all three transgenic lines relative to the wild‐type Col‐0 control (Figure 6C). Besides, we co‐expressed GFP‐SlGSNOR with Myc‐PcRD18, or the control Myc‐GUS, in *N. benthamiana* and determined the protein levels of SlGSNOR in the presence or absence of PcRD18, at 2 dpi. As expected, immunoblots showed that co‐expression with PcRD18 decreased the amounts of the SlGSNOR protein (Figure 6D). In contrast, the SlGSNOR^C10/271/370S^ mutant, (abbreviated as SlGSNORM1), which has lost the *S‐*nitrosylation sites and thus cannot be degraded via the selective autophagy pathway,^[^ ^47^, ^48^ ^]^ remained unaffected by co‐expression with PcRD18 (Figure 6D). This suggests that PcRD18 indeed destabilizes SlGSNOR via the autophagy pathway. To confirm this, the autophagy inhibitor 3‐MA or the 26S proteasome inhibitor MG132 was infiltrated into the leaves at 12 h before sampling. Protein accumulation assays showed that, when compared with the DMSO control treatment, 3‐MA abolished the destabilization of the SlGSNOR protein by PcRD18, while MG132 did not (Figure 6E). Given that *S*‐nitrosylation promotes ATG8 binding to GSNOR during its autophagy‐dependent degradation in Arabidopsis,^[^ ^47^ ^]^ we next questioned whether PcRD18 modulates the SlGSNOR‐ATG8c interaction. To investigate this, we performed a co‐immunoprecipitation analysis, which revealed that PcRD18 significantly enhances the association between GFP‐tagged SlGSNOR and mCherry‐tagged ATG8c (Figure 6F). These results reveal that PcRD18 destabilizes SlGSNOR through the autophagy pathway by facilitating its interaction with ATG8c.

### 2.7 PcRD18 Disturbs the NO Homeostasis and Elevates the Overall Plant *S*‐nitrosylation Levels by Interacting with GSNOR

**Figure 7:**
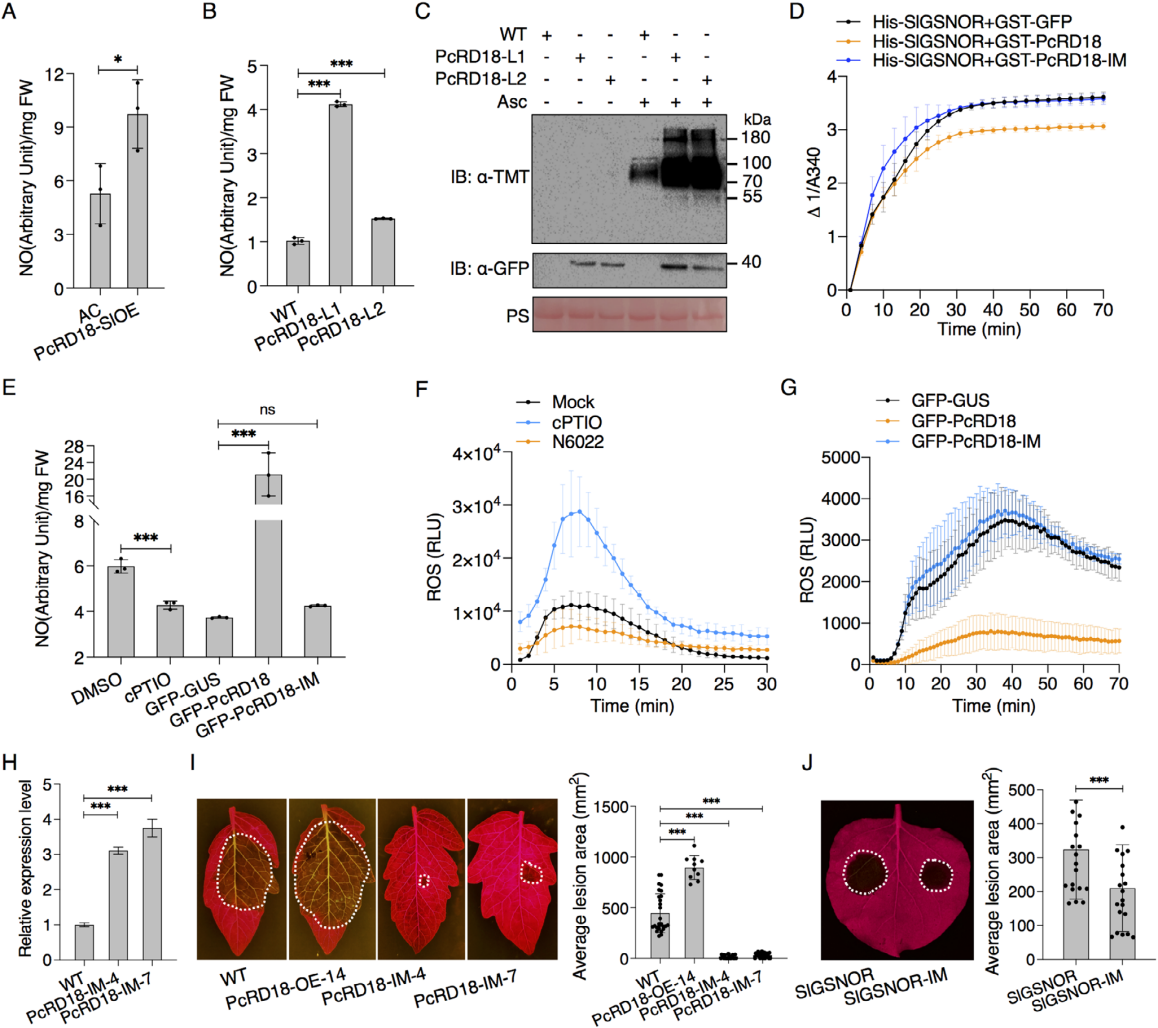
PcRD18 elevates cellular NO and *S*‐nitrosylation levels, dependent on its interaction with GSNOR. Stable expression of PcRD18 in tomato A) and Arabidopsis B) elevates the cellular NO content. The PcRD18‐transgenic tomato line, PcRD18‐SlOE, along with the Arabidopsis lines PcRD18‐L1 and L2, was utilized to measure the cellular nitric oxide (NO) content using DAF‐2 DA, a NO‐sensitive probe. The wild‐type tomato cultivar Ailsa Craig (AC) and Arabidopsis Col‐0 served as respective controls. The fluorescence intensity is expressed in arbitrary units, normalized to milligrams of leaf fresh weight (FW). Statistical analysis was performed with *t*‐tests (mean ± SD; *n* = 3; ^*^, *P* < 0.05; ^***^, *P* < 0.001). C) PcRD18 increases the overall cellular protein *S‐*nitrosylation level. Total proteins were extracted from leaves of 4‐week‐old Arabidopsis plants and a modified *S‐*nitrosylation assay with non‐biological iodoTMT^TM^ reagent for labeling was performed to detect *S‐*nitrosylated proteins. Samples without sodium ascorbate (Asc) treatment were used as negative controls. D) PcRD18‐IM has lost the ability to inhibit the enzymatic activity of SlGSNOR. The enzymatic activity of His‐SlGSNOR was measured in vitro upon co‐incubation with GST‐PcRD18‐IM, GST‐PcRD18 or the control GST‐GFP. The consumption of NADH, as indicated by △1/A340, was used to indicate the relative enzyme activity. Data are presented as mean ± SD (*n* = 3). E) PcRD18‐IM has lost the ability to elevate the cellular NO content. GFP‐PcRD18, GFP‐PcRD18‐IM and GFP‐GUS were expressed in *N. benthamiana* leaves. Treatment with cPTIO was used as a control. *N. benthamiana* leaves were subjected to treatment with 200 µm cPTIO or 0.1% DMSO for 4 h, followed by sampling for NO measurement. NO levels were detected at 2 days post ago‐infiltration (dpi). Statistical analysis was performed with one‐way ANOVA (mean ± SD; *n* = 3; ^***^, *P* < 0.001; ns, not significant). F) Flg22 induced ROS burst on treatment with cPTIO and N6022. *N. benthamiana* leaves treated with 100 µm cPTIO and 500 µm N6022 were used to detect the NO content. Leaves treated with 0.1% DMSO (Mock) were used as a control. G) PcRD18‐IM does not suppress the flg22‐induced ROS burst. GFP‐PcRD18‐IM, GFP‐PcRD18 and the control GFP‐GUS were transiently expressed in *N. benthamiana* leaves after which the ROS burst induced by 10 µM flg22 was measured at 2 dpi. The indicated values represent the means ± SD (n≥3) in F,G). H) Relative expression levels of *PcRD18‐IM* in two independent *PcRD18*‐*IM*‐transgenic lines (PcRD18‐IM‐4 and ‐7) and the wild type (*P. capsici* BYA5 strain). Total RNA was extracted from the mycelium of *P. capsici* cultivated in the liquid medium, and RT‐qPCR analysis was conducted to quantify the transcript levels of *PcRD18‐IM* in each sample. *PcActin* was used for normalization. Data are presented as mean ± SD (*n* = 3). I) Stable expression of *PcRD18‐IM* in *P. capsici* does not enhance its infection of tomato. PcRD18‐overexpression strain (PcRD18‐OE‐14) was used as a control. Detached leaves were inoculated with mycelial plugs of *P. capsici* and photographed at 2 days after inoculation. One‐way ANOVA was used to determine the significant differences between each group and the wild‐type control AC (mean ± SD; n≥10; ^***^, *P* < 0.001). J) Transient expression of *SlGSNOR‐IM* in *N. benthamiana* decreases lesion formation by *P. capsici* compared to wild‐type *SlGSNOR*. *N. benthamiana* leaves were agro‐infiltrated with *GFP‐SlGSNOR‐IM* and *GFP‐SlGSNOR* at the indicated sites, followed by inoculation with *P. capsici* 12 h later. Lesions were then photographed and measured 2 days after‐inoculation. Data are presented as mean ± SD, with asterisks indicating significant differences (*t*‐test; n≥10; ^***^, *P* < 0.001). White dashed lines indicate the lesion areas.

Considering the role of GSNOR in maintaining the NO homeostasis and determining overall protein *S*‐nitrosylation levels,^[^ ^6^, ^49^ ^]^ we hypothesized that PcRD18 might interfere with plant NO homeostasis and overall protein *S‐*nitrosylation levels by manipulating GSNOR abundance and activity. To verify this, we measured the NO content using 4,5‐diaminofluorescein‐2/diacetate (DAF‐2/DA) probe and tested the total protein *S‐*nitrosylation levels by TMT‐switch assay in leaves of 4‐week‐old PcRD18‐L1, ‐L2 and wild type Col‐0 Arabidopsis plants. Our results show that the NO content is elevated in both *PcRD18*‐transgenic tomato and Arabidopsis lines, when compared to their respective wild type (WT) controls, AC and Col‐0 (**Figure** **7**A,B). Western blot analysis, using an anti‐TMT antibody, revealed a significant elevation in the total amount of *S*‐nitrosylated proteins in the PcRD18‐L1 and ‐L2 samples, in comparison to the WT control (Figure 7C). This suggests that PcRD18 disrupts the NO homeostasis and enhances overall protein *S*‐nitrosylation levels in plants.

To investigate whether the virulence function of PcRD18 relies on its binding to SlGSNOR, we assessed the impact of PcRD18‐IM on the enzymatic activity of SlGSNOR. The results revealed that GST‐PcRD18 exhibited a clear suppressive effect on SlGSNOR activity, whereas GST‐PcRD18‐IM, similar to the GST‐GFP control, did not alter the catalytic activity of SlGSNOR toward GSNO (Figure 7D), suggesting that the mutations within the interaction sites of PcRD18 abolished its ability to suppress SlGSNOR activity. To examine whether the mutations affect the regulatory role of PcRD18 in NO homeostasis, we analyzed the NO content in *N. benthamiana* leaves transiently expressing GFP‐PcRD18, GFP‐PcRD18‐IM, and the control GFP‐GUS. After treatment with the NO scavenger carboxy cPTIO (2‐(4‐carboxyphenyl)‐4,5‐dihydro‐4,4,5,5‐tetramethyl‐1H‐imidazolyl‐1‐oxy‐3‐oxide), we observed a decrease in NO content (Figure 7E). Notably, the expression of GFP‐PcRD18 led to a significant increase in NO levels compared to the expression of the GFP‐GUS control, however, the expression of GFP‐PcRD18‐IM did not result in a similar increase in NO content, unlike the expression of GFP‐PcRD18 (Figure 7E), suggesting that the binding to SlGSNOR is essential for PcRD18 to elevate NO levels. The NO scavenger cPTIO and the GSNOR inhibitor N6022 were used as controls in our experiment, and we observed that cPTIO elevated the ROS burst, while N6022 inhibited it (Figure 7F). Consistently, PcRD18‐IM expressed in *N. benthamiana* failed to suppress the ROS burst induced by flg22, in contrast to the suppressive effect that was observed upon expressing PcRD18 (Figure 7G). Western blotting confirmed the correct expression of both PcRD18 and PcRD18‐IM proteins (Figure S14, Supporting Information). To investigate the role of PcRD18 and PcRD18‐IM in a *GSNOR*‐deficient background, we conducted transient expression assays in TRV‐*NbGSNOR*‐inoculated *N. benthamiana* leaves, with TRV‐*GUS*‐inoculated leaves as controls. Subsequent inoculation assays revealed that in TRV‐*GUS* control leaves, transient expression of *PcRD18* significantly increased plant susceptibility to *P. capsici* compared to PcRD18‐IM (Figure S15, Supporting Information). However, in TRV‐*NbGSNOR* leaves, no significant difference was observed between PcRD18 and PcRD18‐IM in altering plant susceptibility (Figure S15, Supporting Information), suggesting that PcRD18 requires host GSNOR to facilitate infection. To further confirm the impact of mutations on the ability of PcRD18 in promoting infection, we genetically transformed the *P. capsici* strain BYA5 with the PcRD18‐IM construct. Two transgenic strains, PcRD18‐IM‐4 and PcRD18‐IM‐7, were obtained and were confirmed to overexpress *PcRD18‐IM* by RT‐qPCR analysis (Figure 7H). Inoculation assays demonstrated that the PcRD18‐IM strains failed to promote infection as compared with the wild‐type strain, while the PcRD18‐OE strain did, indicating that mutations in the interaction sites abolished the virulence function of PcRD18 (Figure 7I). During the transformation process utilizing PcRD18‐IM, we additionally obtained three transgenic lines, namely PcRD18‐RNAi‐2, PcRD18‐RNAi‐3, and PcRD18‐RNAi‐9, in which the *PcRD18* gene was effectively silenced (Figure S16A, Supporting Information). When these PcRD18‐RNAi strains were inoculated onto tomato leaves, the results demonstrated that silencing of *PcRD18* significantly diminished the virulence of *P. capsici* (Figure S16B, Supporting Information), further confirming the crucial role of PcRD18 in determining *P. capsici* virulence. These results indicate that the virulence function of PcRD18 is dependent on its binding to SlGSNOR. Therefore, we hypothesized that mutating specific sites on GSNOR may enhance plant resistance by disrupting the interaction with the effector protein PcRD18. Consistent with this hypothesis, transient expression of SlGSNOR‐IM, in which these sites were mutated, resulted in decreased lesion formation by *P. capsici* when compared to wild‐type SlGSNOR (Figure 7J). This phenotype was consistently observed in TRV‐*NbGSNOR* plants having a reduced GSNOR background (Figure S17, Supporting Information). Furthermore, AlphaFold structural predictions showed equivalent folding patterns and secondary structures when comparing SlGSNOR‐IM and wild‐type SlGSNOR (Figure S18A, Supporting Information). Besides, SlGSNOR‐IM effectively decomposed its substrate GSNO and reduced cellular NO levels (Figure S18B,C, Supporting Information), indicating that SlGSNOR‐IM is still active. Additionally, SlGSNOR‐IM enhanced the INF1‐induced cell death (Figure S18D,E, Supporting Information), mirroring the immune‐enhancing role of wild‐type SlGSNOR. These data collectively indicate that SlGSNOR‐IM preserves its native structure and function, while evading PcRD18 targeting. Notably, the sites on GSNOR that are targeted by PcRD18 are conserved across different plant species (Figure S19, Supporting Information), providing a common reference for plant resistance improvement strategies.

## 3 Discussion

**Figure 8:**
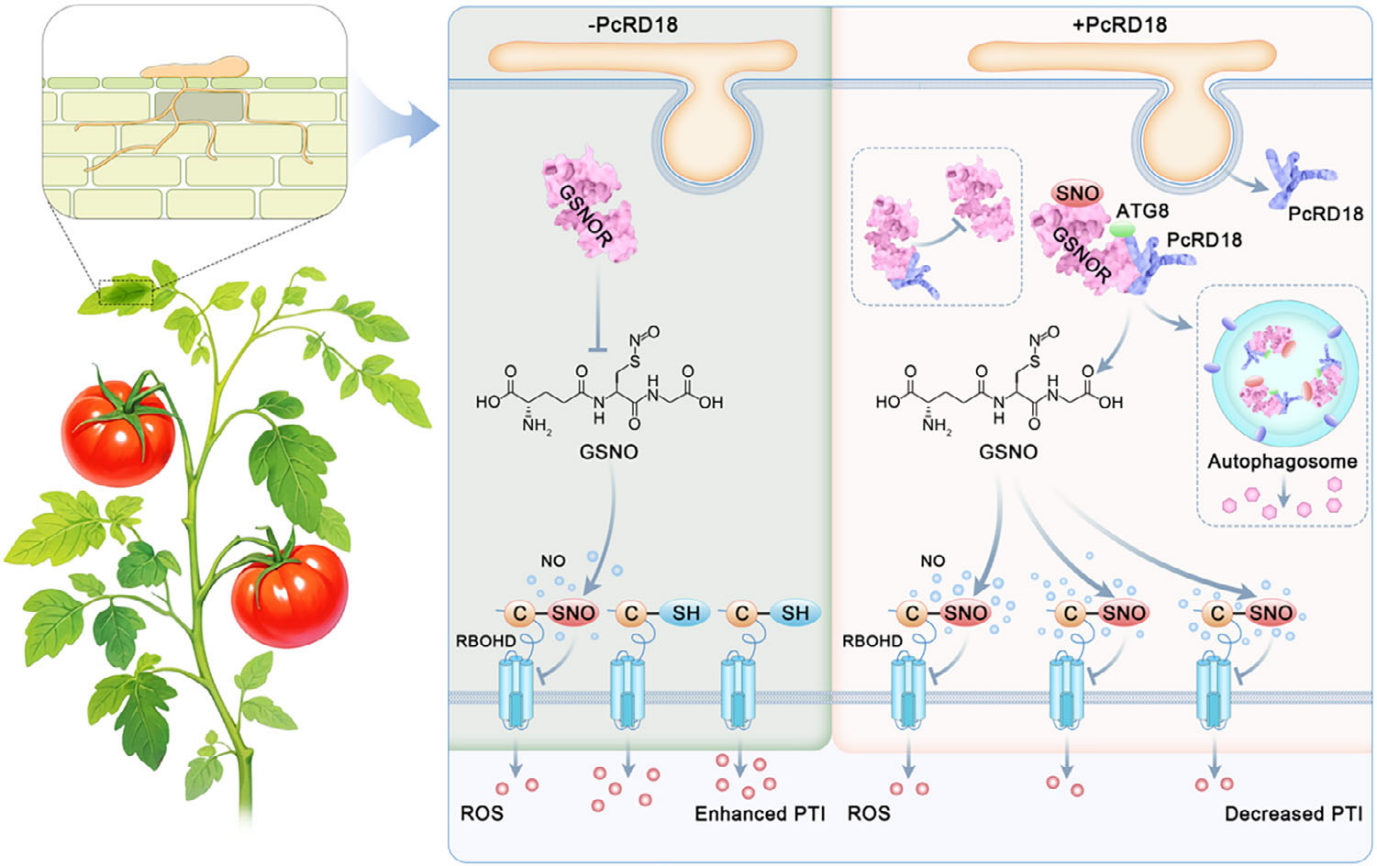
PcRD18 subverts plant immunity by hijacking NO homeostasis through targeting of GSNOR. GSNOR, a key enzyme maintaining NO homeostasis by degrading GSNO, functions as a “security lock” to prevent aberrant *S*‐nitrosylation and ensure proper immune signal transduction.^[^ ^6^, ^21^, ^45^ ^]^ Upon *P. capsici* infection, GSNOR inhibits GSNO accumulation, thereby restricting *S*‐nitrosylation (SNO) of immune‐related proteins such as RBOHD, ensuring sufficient enzymatic activity for ROS production^[^ ^16^ ^]^ and thus enhancing plant defense (left panel). However, *P. capsici* has evolved the RxLR effector PcRD18 to disrupt this balance. PcRD18 interacts with GSNOR, both inhibiting its enzymatic activity and promoting its degradation via the autophagy pathway by facilitating its interaction with ATG8. This “unlocking” of GSNOR by PcRD18 leads to elevated NO levels and increased global protein *S*‐nitrosylation, particularly of immune‐related proteins like RBOHD. The enhanced *S*‐nitrosylation of RBOHD inhibits its activity,^[^ ^16^ ^]^ resulting in a decreased ROS burst and a compromised PTI (right panel). Our findings reveal a pathogen strategy to disrupt plant redox signaling by effector‐mediated hijacking of the central NO regulatory enzyme GSNOR. These findings offer mechanistic insights into host‐pathogen redox dynamics and provide possible strategies for enhancing plant resistance through effector target engineering that should circumvent pathogen manipulation.

Protein *S‐*nitrosylation plays important roles in plant defense. Whether pathogen effectors manipulate plant *S‐*nitrosylation modification of cellular proteins to suppress host defense has remained unknown. Here, we identified the RxLR effector PcRD18 that promotes *P. capsici* infection (Figure 1), suppresses the PAMP induced ROS burst, inhibits MAPK activation, and suppresses plant cell death and SA‐related *PR* gene expression (Figure 2). Especially, the flg22‐induced ROS burst is nearly abolished by PcRD18. ROS production is a hallmark of successful activation of plant defenses and plays a crucial role in immune signal transduction.^[^ ^50^, ^51^ ^]^ Therefore, PcRD18 is a key virulence effector of *P. capsici*. We provide evidence that PcRD18 targets and inhibits host GSNOR to elevate the cellular NO content and overall protein *S‐*nitrosylation levels, thereby interfering in the activity of key regulators of PTI responses, especially the ROS burst, to thereby promote host susceptibility. In **Figure** **8**, we propose a working model for the role of PcRD18 in virulence of *P. capsici*. Moreover, we predicted the key interaction sites between PcRD18 and GSNOR (Figure 3) and mutation of these sites in PcRD18 (PcRD18‐IM) resulted in loss of its ability to target and suppress GSNOR, as well as its ability to disturb the NO homeostasis, inhibit the ROS burst and promote infection (Figure 7D–I). PcRD18‐IM‐ transformed strains of *P. capsici* show lower levels of virulence than the wild‐type strain, indicating a dominant negative effect^[^ ^52^, ^53^ ^]^ of PcRD18‐IM on PcRD18. These findings indicate that the role of PcRD18 in virulence depends on its manipulation of GSNOR. Our study sheds light on a novel infection strategy, by which pathogens manipulate host *S‐*nitrosylation modification to suppress a series of plant immune responses. Uncovering the sites of SlGSNOR targeted by PcRD18 may provide insight for precisely improving plant immunity by assisting plants in defending against pathogen targeting.

### 3.1 GSNOR Positively Regulates Plant Immunity to *Phytophthora* Pathogens

GSNOR positively regulates plant immunity to *Phytophthora* pathogens as the tomato lines overexpressing *SlGSNOR* showed an enhanced resistance to *P. capsici* (Figure 4A,B), while silencing or knocking out of *SlGSNOR* decreased the resistance levels (Figure 4C,D). Transient expression of *CaGSNOR* (Figure S9A, Supporting Information) or inhibition of *CaGSNOR* enzymatic activity (Figure S9B, Supporting Information) in pepper and transient expression of *SlGSNOR* (Figure S10A, Supporting Information) or silencing of *NbGSNOR* (Figure S10B, Supporting Information) in *N. benthamiana* also confirmed that GSNOR positively regulates plant defense against *P. capsici*. These results are consistent with the role of AtGSNOR in Arabidopsis defense against *P. syringae*, *H. parasitica*, and *P. parasitica* ^[^ ^49^, ^54^ ^]^ and of SlGSNOR in tomato defense against *P. syringae*.^[^ ^38^ ^]^ Mutations in the active site of SlGSNOR abolished its role in resistance to *P. capsici* (Figure 4E,F), suggesting that the enzymatic activity of SlGSNOR is required for its function in plant defense.

### 3.2 PcRD18 Modulates Host Protein *S‐*nitrosylation Modification via Inhibiting GSNOR

It has been reported that GSNOR regulates the *S‐*nitrosylation of proteins through the reduction of GSNO, which is a major trans‐nitrosylation molecule in the cellular machinery.^[^ ^3^, ^4^, ^21^, ^39^ ^]^ Protein *S‐*nitrosylation plays an important role in plant‐pathogen interactions, as especially the activity of several key regulators in plant immunity is regulated by *S‐*nitrosylation modification, including RBOHD, APX, NPR1, SABPs and BOTRYTIS‐INDUCED KINASE 1 (BIK1).^[^ ^16^, ^17^, ^19^, ^20^, ^55^ ^]^ Consequently, modulation of host protein *S‐*nitrosylation levels would be an important strategy used by pathogens to colonize host plants. Studies showing that Arabidopsis GSNOR activity is suppressed by secreted proteins from *P. parasitica*,^[^ ^54^ ^]^ and that the amount of tomato protein *S‐*nitrosothiols is increased after infection by *P. infestans* and *P. capsici*,^[^ ^56^ ^]^ already suggested that pathogens are able to modulate host *S‐*nitrosylation levels to facilitate colonization. However, whether pathogens actually utilize effectors to manipulate host *S‐*nitrosylation remained unknown. Here we show that PcRD18 suppresses the enzymatic activity and accumulation of SlGSNOR (Figure 6A–C), thereby elevating the NO content and *S‐*nitrosylation levels in the host cells (Figure 7A–C). *S‐*nitrosylated GSNOR was reported to be degraded via the autophagy pathway.^[^ ^47^ ^]^ Considering that PcRD18 elevates the cellular *S‐*nitrosylation levels, it is likely that the destabilization of GSNOR by PcRD18 is due to the increased overall *S‐*nitrosylation modification of proteins in the host cells. By introducing mutations in the sites of GSNOR that can be *S*‐nitrosylated or by adding the autophagy inhibitor 3‐MA, GSNOR becomes no longer vulnerable to destabilization by PcRD18 (Figure 6D,E). Moreover, our results indicate that PcRD18 largely facilitates the interaction between SlGSNOR and ATG8c (Figure 6F), and this interaction is shown to be enhanced upon *S*‐nitrosylation of GSNOR.^[^ ^47^ ^]^ These results further support the observation that increased *S‐*nitrosylation modification by PcRD18 leads to GSNOR degradation via the autophagy pathway. Our research provides an example of the manipulation of overall plant *S‐*nitrosylation modification of cellular proteins by the pathogen effector PcRD18, via inhibiting its host target GSNOR.

### 3.3 PcRD18 Suppresses Immunity by Elevating *S‐*nitrosylation of Key Components Involved in PTI

It is known that both RBOHD and APX, playing key roles in ROS production and detoxification, respectively,^[^ ^57^, ^58^, ^59^, ^60^, ^61^ ^]^ are both regulated by *S‐*nitrosylation modifications.^[^ ^16^, ^17^ ^]^ Overexpressing AtGSNOR results in an increased RBOHD activity and a decreased APX activity, leading to an increased accumulation of ROS in the cell.^[^ ^16^, ^17^ ^]^ Besides, increased *S‐*nitrosylation of NPR1 and SABP, which are two key regulators of SA signaling,^[^ ^62^ ^]^ suppresses their functions and hence curbs the SA defense.^[^ ^19^, ^20^ ^]^ These observations support our results showing that GSNOR positively regulates the flg22‐induced ROS burst and the SA‐related *PR* gene expression (Figure 5). The mechanism by which GSNOR regulates the flg22‐induced MAPK activation, and the INF1‐triggered cell death currently remains unknown. Possibly, the functions of upstream RLKs or RLCKs that are involved in MAPK activation or INF1‐induced cell death are regulated by *S‐*nitrosylation. Consistent with the fact that PcRD18 suppresses various immune responses, i.e., the ROS burst, MAPK activation, *PR* gene expression, and INF1‐induced cell death, all of which are positively regulated by GSNOR (Figure 2), the PcRD18‐IM mutant indeed lost the ability to modulate SlGSNOR activity, NO homeostasis, immune response and to promote infection (Figure 7D–I). This further indicates that PcRD18 functions as a virulence effector by targeting and inhibiting SlGSNOR, which plays a pivotal role in regulating the *S*‐nitrosylation of key components involved in PTI. Consistent with this, when the sites on SlGSNOR targeted by PcRD18 were mutated, the mutated SlGSNOR (SlGSNOR‐IM) exhibited enhanced resistance to *P. capsici* (Figure 7J), as these mutations may enable SlGSNOR to evade the inhibitory effects of the effector protein PcRD18. Moreover, structure predictions and functional assays confirmed that SlGSNOR‐IM retains the native structure, enzymatic activity, and immune functions of wild‐type SlGSNOR (Figure S18, Supporting Information). We note, however, that high‐resolution structural validation through techniques such as X‐ray crystallography or cryo‐electron microscopy will be important to exclude local conformational changes. Notably, the sites on GSNOR targeted by PcRD18 are conserved across different plant species (Figure S19, Supporting Information), highlighting the potential of these mutations for improving plant resistance through targeted genetic modifications. Future studies should aim to identify minimal sets of mutations that block PcRD18 binding while preserving the native structure and enzymatic activity of GSNOR through a stepwise interface reduction strategy. Following biophysical binding assays of the mutants, enzymatic activity assays combined with inoculation assays, will validate whether engineered variants retain their catalytic function and immune‐enhancing capacity. These strategies may facilitate the development of broad‐spectrum resistance against *Phytophthora* pathogens in crops.

Collectively, we propose a working model for the RxLR effector PcRD18 in suppressing host immunity. In response to *P. capsici* infection, plants activate GSNOR to lower the GSNO levels and thereby repress the *S*‐nitrosylation of immune regulators, for example RBOHD, to activate the ROS burst to hamper infection. However, *P. capsici* secretes the RxLR effector PcRD18, which binds to GSNOR, and thereby inhibits its enzymatic activity and lowers its stability. The binding of PcRD18 to GSNOR results in enhanced *S*‐nitrosylation levels in the plant cells, which enhance the *S*‐nitrosylation of immune regulators, such as RBOHD, to hamper the ROS burst and other immune responses (Figure 8). By gaining an in‐depth understanding of how pathogens interfere with host *S*‐nitrosylation, our study provides a novel view on the plant‐pathogen interface and provides leads for new strategies aimed at improving plant resistance to pathogens.

## 4 Experimental Section

### Plant and Microbe Cultivation

*N. benthamiana*, Arabidopsis and tomato plants were grown in a growth chamber at 22 °C with long‐day conditions of 16‐h light/8‐h dark. *P. capsici* strains LT263 and BYA5 were cultivated in 25 °C incubators on carrot agar (CA) or V8 medium. *Agrobacterium tumefaciens* strains C58C1 and GV3101 were grown at 28 °C on Luria‐Bertani (LB) medium. *Escherichia coli* DH5α and BL21were routinely cultured in LB medium at 37 °C.

### Plasmid Construction

For expression constructs, coding regions of the genes were cloned into a pART27 vector. For generating VIGS constructs, the cDNA fragments designed by the VIGS tool^[^ ^63^ ^]^ were cloned into the pBinary tobacco rattle virus (TRV2) vector. pCAMBIA‐Nluc and pCAMBIA‐Cluc were used for luciferase complementation assays. The vectors PGEX‐6p‐1 with a GST tag and PET32a with a His tag were used for production of recombinant proteins in the *E. coli* strain BL21. To generate the non‐nitrosylatable mutant SlGSNORM1, the conserved C10, C271, and C370 residues of SlGSNOR were selected for mutagenesis, as previous evidence indicated that these sites undergo *S*‐nitrosylation.^[^ ^48^ ^]^ Furthermore, this modification has been shown to trigger autophagy‐dependent degradation of GSNOR.^[^ ^47^ ^]^ To create this mutant, site‐directed mutagenesis PCR was performed of the coding region of *SlGSNOR*, changing each codon for the conserved cysteine residue to a codon for serine (C10S/C271S/C370S).

### Agrobacterium‐Mediated Transient Expression in *N. benthamiana*

The *Agrobacterium* strain GV3101 or C58C1, carrying the various constructs, were cultured in LB liquid medium with the appropriate antibiotics at 28 °C for 16–24 h. After being pelleted, the bacteria were resuspended in an infiltration buffer containing 10 mm MES, 10 mm MgCl_2_, and 200 mm acetosyringone. Agrobacteria suspensions driving expression of the appropriate proteins were mixed and adjusted to the required OD_600_ before infiltration.

### VIGS in *N. benthamiana* and Tomato

The 300–500 bp fragments of *NbGSNOR* or *SlGSNOR* designed by the VIGS tool were amplified by PCR using the primers indicated in Table S2 (Supporting Information) and were inserted into the TRV2 vector. A TRV construct containing a fragment of the *GUS* gene was used as a negative control. Cultures of *Agrobacterium* strain GV3101 containing the TRV1 and TRV2 vectors were mixed and infiltrated into the leaves of four‐leaf–stage *N. benthamiana* plants, at a final OD_600_ of 0.3. Plants infiltrated with TRV‐*PDS*, containing a fragment of the gene encoding phytoene desaturase, were used as a control to monitor the silencing progress, as these plants eventually showed photobleaching. Gene silencing efficiency was determined by RT‐qPCR analysis at 3–4‐week after agro‐infiltration.

### Transformation of *P. capsici*

For overexpressing *PcRD18* and *PcRD18‐IM* in *P. capsici*, the full‐length coding sequence of *PcRD18* or *PcRD18‐IM* was cloned into the pYF3 vector containing the ham34 promoter and ham34 terminator and subsequently transformed into *P. capsici* strain BYA5. Transformants were generated through polyethylene glycol (PEG)‐CaCl_2_‐mediated protoplast transformation and subsequently screened on V8 medium supplemented with the selective antibiotic G418 (50 µg/mL).^[^ ^64^ ^]^

### Phytophthora Inoculation Assay

*P. capsici* strains were cultured on CA plates at 25 °C. To inoculate *N. benthamiana* and tomato plants with mycelium, the leaves were incubated with mycelial plugs for 1.5–2 days. For zoospore inoculation assays, the agar with the mycelium was then cut into 0.5 cm squares and placed in CA liquid medium for 2–3 days. The medium was replaced by fresh water every two days, up to three times to produce sporangia. Zoospores were released with a cold shock at 4 °C for 1 h, followed by a 1 h equilibration at room temperature. The detached leaves of 4–6‐week‐old *N. benthamiana* plants, 4–8‐week‐old tomato and 4‐week‐old Arabidopsis plants were inoculated with the *P. capsici* zoospore suspension (400–800 zoospores per inoculation site). The size of the lesions that developed on the leaves were measured at 2–3 days after inoculation.

### Oxidative Burst Measurement

A luminol‐based chemiluminescence assay was used for ROS burst measurements. Leaf discs were taken from *N. benthamiana* transiently expressing GFP‐PcRD18 or the control GFP‐GUS, at 2 days post agro‐infiltration, and incubated with ddH_2_O overnight. The leaf discs were subsequently transferred to the reaction mixture containing 20 µm luminol and 10 µg mL^−1^ horseradish peroxidase, in 96‐well plates, after which a multimode microplate reader (Thermo Scientific Varioskan LUX, USA) was used to measure the luminescence of luminol over a period of 30–40 min after addition of 10 µm flg22.

### Plant Cell Death Assay

Cell death was induced by expression of the apoplastic *P. infestans* PAMP protein INF1 in *N. benthamiana*. The percentage of the area showing cell death as part of the total infiltrated area was divided into three levels (0%, 0%–50%, and 50%–100%) as previously described^[^ ^65^ ^]^ and analyzed by Wilcoxon rank‐sum tests. Ion leakage analyses were performed as described before.^[^ ^66^ ^]^ Briefly, the leaf discs punched from infiltrated areas were floated on sterile distilled water, followed by shaking at 200 rpm for 2 h. The conductivity of the water was then measured before and after boiling the samples for 5 min. Relative ion leakage was calculated as the percentage of the conductivity before compared to that after boiling.

### RNA Isolation and RT‐qPCR Analysis

RNA was extracted using the Plant RNA Isolation Mini Kit (Bioteke, Cat.RP3301). First‐strand cDNA was synthesized from total RNA with the Evo M‐MLV RT Mix Kit, with gDNA Clean for qPCR (Accurate Biotecknology, Cat.AG11728). Gene transcript expression levels were determined by reverse transcription quantitative real‐time PCR (RT‐qPCR) analysis, using SYBR green Supermix (Tsingke, Cat. TSE401) with gene‐specific primers (Table S2, Supporting Information).

### Identification of PcRD18‐Interacting Proteins by Immunoprecipitation Coupled with Mass Spectrometry

Total proteins extracted from *N. benthamiana* transiently expressing GFP‐PcRD18 at 2 days after infiltration were incubated with GFP‐trap_A beads (Chromotek, Planegg‐Martinsried, Germany) at 4 °C for 3 h. The beads were then washed with dilution buffer consisting of 10 mm Tris‐HCl (pH 7.5), 150 mm NaCl, and 0.5 mm EDTA, for five times. The obtained protein complexes, enriched by the GFP‐beads were pretreated as described earlier^[^ ^67^ ^]^ and then submitted to high‐sensitivity LC‐MS/MS (QExactive HF‐X, ThermoFisher, Waltham, MA, USA) analysis.

### Luciferase Complementation Assay

Agrobacterium containing Cluc‐ and Nluc‐fused constructs were co‐infiltrated into *N. benthamiana* leaves and after 2 days leaves were sprayed with luciferin (Biovision) and kept in the dark for 5 min, as previously described.^[^ ^68^ ^]^ The images were subsequently captured by a CCD‐camera.

### Co‐immunoprecipitation Assay

Agrobacterium cultures were infiltrated into leaves of 6‐week‐old *N. benthamiana* plants and total proteins were extracted 2 days later, using lysis buffer containing 10% glycerol, 25 mm Tris‐HCl (pH 7.5), 1 mm EDTA, 150 mm NaCl, 0.15% [v/v] NP‐40, 1mM DTT, 2% (w/v) polyvinylpolypyrrolidone (PVPP) and protease inhibitor cocktail (Biotool, USA). After centrifugation at 4 °C for 30 min (15, 000 × g), 1.5 mL of the supernatant was incubated with 15 µL of GFP‐Trap beads (Chromotek, Germany) at 4 °C for 3 h. The beads were collected by centrifugation (2, 500 x g) and washed seven times using washing buffer (10% glycerol, 25 mm Tris‐HCl (pH 7.5), 1 mM EDTA, 150 mm NaCl, 0.15% [v/v] NP‐40, 1 mM DTT). Bound proteins were obtained by incubating the beads in a loading buffer at 95 °C for 10 min.

### Glutathione‐S‐transferase (GST) Pull‐Down

Constructs for production of recombinant GST‐PcRD18, GST‐GFP, and His‐SlGSNOR were introduced into *E. coli* strain BL21. Recombinant protein expression and purification were performed as described before.^[^ ^36^, ^69^ ^]^ GST‐PcRD18 and GST‐GFP were purified using glutathione beads 4FF (Smart‐Lifesciences, Cat: SA010025) according to the manufacturer's protocol. His‐SlGSNOR was purified using Pierce Ni‐NTA beads 6FF (Smart‐Lifesciences, Cat: SA005025), according to the manufacturer's protocol. Equal amounts of recombinant GST‐ and His‐tagged proteins were incubated with 50 µL pre‐washed glutathione beads at 4 °C for 2 h. The beads were collected ad washed for five times with washing buffer (25 mm Tris‐HCl, 100 mm NaCl, 1 mm DTT, pH 7.5). His‐tagged and GST‐tagged proteins were detected by immuno‐blotting using anti‐His (ABclonal, Cat: AE003) and anti‐GST (ABclonal, Cat: AE006) antibodies.

### Western Blot Assays

Total proteins were extracted using ice‐cold lysis buffer (25 mM Tris‐HCl pH 7.4, 150 mm NaCl, 1 mM EDTA, 0.15% NP‐40), supplemented with 2% PVPP, 1 mm DTT and protease inhibitors. Proteins were separated on 8%–10% SDS‐polyacrylamide gels and subsequently transferred to PVDF membranes (Roche). Membranes were probed with primary antibodies overnight at 4 °C. The antibodies used in this study include anti‐GFP (#AE011, ABclonal), anti‐Myc (#AE009, ABclonal), anti‐GST (#AE006, ABclonal), anti‐His (#AE003, ABclonal), anti‐GSNOR (#PHY0203A, PhytoAB) and anti‐mCherry (#AE002, ABclonal). Following three TBST (Tris Buffered Saline with Tween 20) washes, membranes were incubated with HRP‐conjugated secondary antibodies for 1–2 h at room temperature. eECL substrate (CWBIO, Beijing, China) was utilized for signal detection.

### Generation of Transgenic Plants

For Arabidopsis transformation, *Agrobacterium* GV3101 carrying GFP‐PcRD18 in plasmid pART27 was cultured in LB liquid medium with the appropriate antibiotics. The bacterial cells were collected by centrifuging for 5 min at 3500 × g and re‐suspended in a 5% sucrose solution containing 0.02% Silwet L‐77, at a final density of OD_600_ = 0.8. Arabidopsis flower buds were dipped in the Agrobacterium suspension for 15 s, with gentle agitation. The plants were then kept in a humid and dark environment for 12 h. The seeds of dipped Arabidopsis were then plated on half‐strength Murashige and Skoog (MS) plates containing kanamycin to select for transformants. The GFP‐PcRD18 or GFP‐SlGSNOR‐pART27 construct was transformed to tomato (cv. Ailsa Craig, AC) as previously described,^[^ ^70^ ^]^ with some minor modification. Briefly, fully unfolded tomato cotyledons were cut into small segments and placed on MS0 medium (MS medium with 1.5 mg L^−1^ zeatin and 0.5 mg L^−1^ IAA) for 3 days. Agrobacterium GV3101 carrying GFP‐SlGSNOR‐ pART27 was cultured in LB liquid medium with the appropriate antibiotics and centrifuged at 3500 × g for 20 min. The bacterial cell pellets were re‐suspended in liquid MS0 medium containing 100 µm acetosyringone to an OD_600_ = 0.6 and then used for dipping the cotyledons segments. After co‐cultivation for 2 days in the dark, the dipped explants were transferred to MS1 medium (MS medium with 2 mg L^−1^ zeatin, 300 mg L^−1^ timentin, 50 mg L^−1^ kanamycin) for the induction of callus formation. The MS1 medium was replaced every three weeks until callus had been formed. MS2 medium (MS medium with 0.2 mg L^−1^ zeatin, 300 mg L^−1^ timentin, 50 mg L^−1^ kanamycin) was subsequently used for shoot induction, after which MS3 medium (MS medium with 0.2 mg L^−1^ IAA, 300 mg L^−1^ timentin, 50 mg L^−1^ kanamycin) was used for root induction. Rooted plants were transferred to sterile soil in pots and subjected to further analysis.

### GSNOR Activity Assay

For the in vitro GSNOR activity assays, the recombinant proteins His‐SlGSNOR, His‐SlGSNORM3, GST‐PcRD18 and GST‐GFP were expressed in *E. coli* and purified. Enzymatic activity was determined by monitoring the absorbance of NADH at 340 nm as described previously.^[^ ^49^ ^]^ Protein samples containing 40 µg of His‐tagged proteins and 80 µg of GST‐tagged proteins were added to 200 µL of enzyme reaction mix (20 mm Tris HCl, pH 8.0; 0.2 mM NADH; 0.5 mM EDTA) and the reaction was started by adding GSNO at a final concertation of 300 µm. For the in vivo activity assays, total proteins of four‐week‐old Arabidopsis plants were extracted with lysis buffer containing 20% glycerol, 50 mm HEPES (pH 8), 10 mm MgCl_2_, 1 mm EDTA, 1 mm EGTA and protease inhibitor cocktail (Biotool, USA). About 150 µg of total proteins were added to 200 µL of enzyme reaction mix containing 20 mM Tris‐HCl (pH 8), 0.2 mm NADH, and 0.5 mm EDTA. The absorbance of NADH at 340 nm was measured after adding GSNO at a final concentration of 400 µm. Relative enzymatic activity is indicated as △1/A340.

### Spectrofluorometric Detection of NO

To determine the NO content of leaf material, a spectrofluorimetric method was used as previously described.^[^ ^71^ ^]^ Briefly, samples of leaves of Arabidopsis or *N. benthamiana* were ground to a fine powder in liquid nitrogen and the powder was extracted with extraction buffer containing 50mm Tris‐HCl (pH 7.8), 0.1 mm EDTA, 5 mm DTT, 0.2% Triton X‐100, 10% glycerol and 2% PVPP. After centrifuging at 25, 000 × g for 25 min at 4 °C, the supernatant was incubated with diaminofluorescein‐2 diacetate (DAF‐2 DA) at a final concentration of 10 µm, in the dark at 37 °C for 2 h. Fluorescence was measured at excitation and emission wavelengths of 485 and 538 nm with a Thermo Scientific Varioskan LUX Multimode Microplate Reader.

### Determination of Total Protein *S*‐nitrosylation Level

Total proteins were extracted from leaves of 4‐week‐old PcRD18 transgenic Arabidopsis lines PcRD18‐L1, L2 and the wild type Col‐0, using HENS buffer containing 100 mM HEPES (pH 7.8), 1 mm EDTA, 0.1 mm neocuproine, 1% SDS and protease inhibitor cocktail (Biotool, USA). A modified *S‐*nitrosylation switch assay using the Pierce *S‐*Nitrosylation Western Blot Kit (Thermo, USA) was performed following the manufacturer's protocol, to detect *S‐*nitrosylated proteins. In brief, the extracted proteins were treated with 20 mM Methyl Methanethiosulfonate (MMTS) for 30 min at room temperature to block free cysteine thiols. The MMTS was then removed by adding pre‐chilled (−20 °C) acetone and freezing at −20 °C for 2 h. The samples were then treated with sodium ascorbate to reduce the *S‐*nitrosylated cysteines into free cysteine thiols, which were subsequently labeled by the non‐biological iodoTMT reagent. Samples without sodium ascorbate treatment were included as negative controls to rule out incomplete blocking of the free cysteine thiols by MMTS.

### Statistical Analysis

Data are expressed as the mean ± standard deviation (SD). The sample sizes (n) utilized for each statistical analysis are provided in detail in the respective figure legends. Differences in colony diameters, lesion areas, and nitric oxide contents were analyzed with Student's *t*‐tests or one‐way ANOVA; differences in the cell death levels were analyzed with Wilcoxon rank‐sum tests or Kruskal‐Wallis tests. Statistical analyses were conducted using GraphPad Prism 10 or R softwares. Experiments were independently repeated two or three times to ensure reproducibility.

## Conflict of Interest

The authors declare no conflict of interest.

## Author Contributions

T.L. and J.K. equally contributed to this work. Y.D., T.L., J.L., and L.C. conceived the project. J.K., T.L., and H.Z. performed the experiments. L.W. participated in constructing the PcRD18 transgenic tomato plans. Y.D., T.L., J.K., and H.Z. analyzed the data. T.L. and Y.D. wrote the manuscript with the help of all authors. M.H.A.J.J. and M.L. revised the manuscript.

## Supporting information



Supporting Information

Supporting Information

## Data Availability

The data that support the findings of this study are available in the supplementary material of this article.
