# Supplementary material for: Phytophthora Disrupts Plant Immunity by Manipulating Nitric Oxide Homeostasis Through GSNOR Inhibition
Source: Adv Sci (Weinh). 2025 Jun 20;12(33):e03633. doi: 10.1002/advs.202503633 (PMC12412504; doi:10.1002/advs.202503633)
Supplement: Supplementary file 1 — Supporting Information [file ADVS-12-e03633-s001.docx]

Supporting Information

***Phytophthora* Disrupts Plant Immunity by Manipulating Nitric Oxide Homeostasis through GSNOR Inhibition**

Tingting Li^1#^, Jing Kang^1#^, Haizhu Zhang^1^, Lina Wang^1^, Minghui Lu^1^, Lin Cai^2,^*, Jianming Li^1,^*, Matthieu H. A. J. Joosten^3^, Yu Du^1,^*


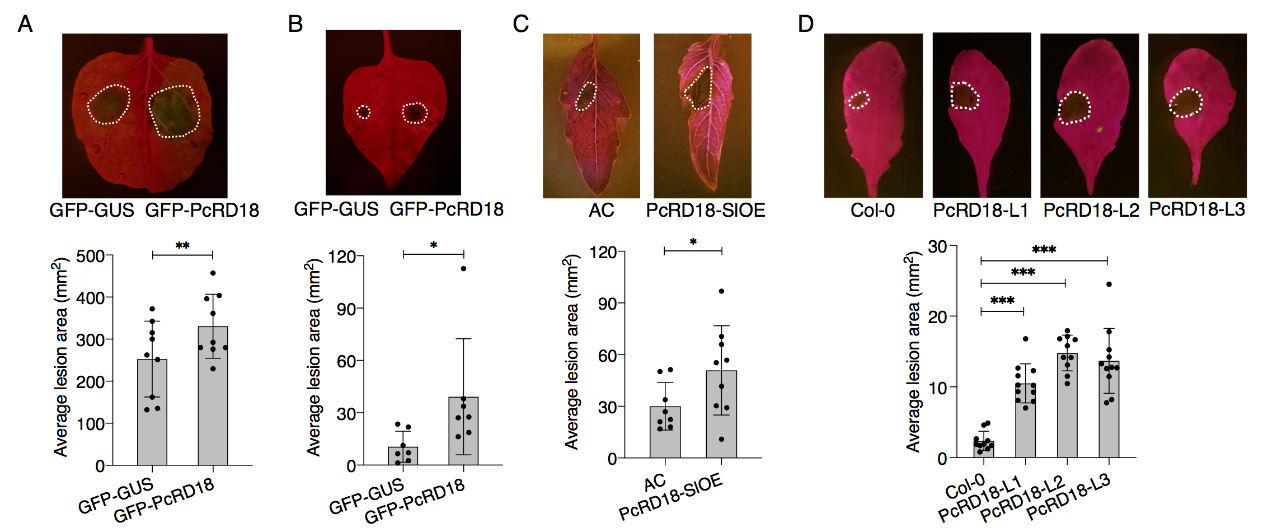


**Figure S1**. Transient and stable expression of PcRD18 promotes host susceptibility to *P. capsici*. Transient expression of PcRD18 enhances *N. benthamiana* **(A)** and pepper **(B)** susceptibility to *P. capsici*. GFP-PcRD18 and the control GFP-GUS were agro-infiltrated into the indicated site of *N. benthamiana* leaves. At 6 hours post infiltration (hpi), the leaves were inoculated with *P. capsici*. The lesions were subsequently photographed and measured at 2 days after inoculation (dai). Pepper leaves transiently expressing GFP-PcRD18, and the control GFP-GUS, were inoculated with *P. capsici* at 24 hpi. The average lesion areas in are shown as the bar graph and was analyzed by paired *t*-tests (mean ± SD; n≥7; *, *P* < 0.05; **, *P* < 0.01) in (**A**, **B**). Stable expression of GFP-PcRD18 in both tomato **(C)** and Arabidopsis **(D)** enhances the susceptibility to *P. capsici*. PcRD18-SlOE is a stable GFP-PcRD18-transgenic tomato line and wild-type tomato Ailsa Craig (AC) was used as a control in (**C**). PcRD18-L1, PcRD18-L2 and PcRD18-L3 are three independent Arabidopsis transgenic lines that stably express GFP-PcRD18 in (**D**). Statistical analysis in (**C**, **D**) was performed with *t*-tests (mean ± SD; n≥8; *, *P* < 0.05; ***, *P* < 0.001). White dashed lines indicate the lesion areas. Each experiment was performed more than two times, with similar results.


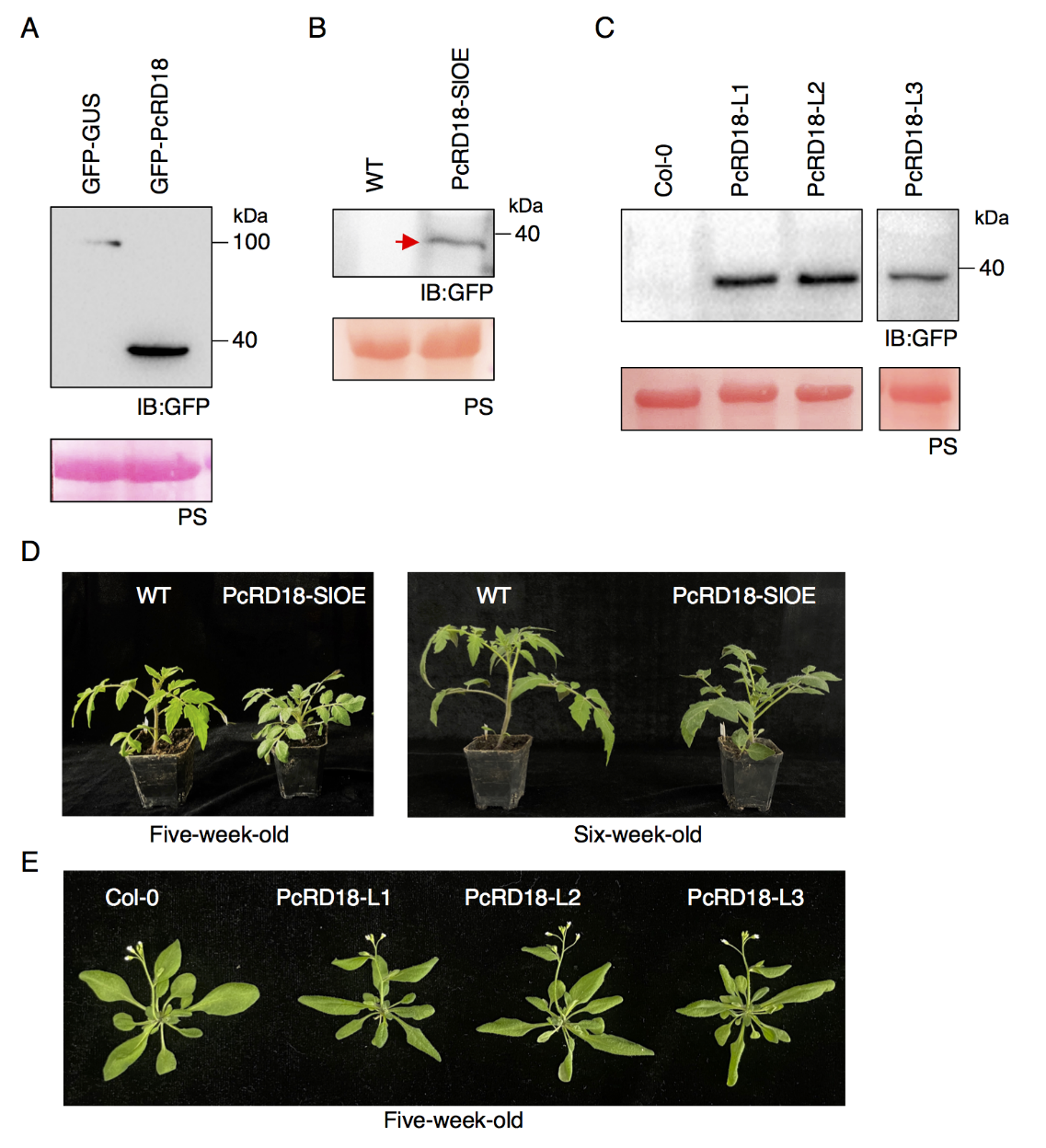


**Figure S2**. Detection of GFP-PcRD18 by immunoblotting and the morphology of *PcRD18*-transgenic Arabidopsis plants. **(A)** Immune-detection of GFP-GUS and GFP-PcRD18 that are transiently expressed in *N. benthamiana*. Proteins were isolated from leaves of *N. benthamiana* transiently expressing GFP-GUS or GFP-PcRD18 at 2 days after agro-infiltration. The predicted molecular weights of GFP-GUS and GFP-PcRD18 are about 100 and 40 kilodaltons (kDa), respectively. Accumulation of GFP-PcRD18 in the *PcRD18*-transgenic tomato plant PcRD18-SlOE, as indicated by the red arrow **(B)** and in the transgenic Arabidopsis lines PcRD18-L1/L2/L3 **(C)**. Leaves of four-week-old tomato and Arabidopsis plants were used for total protein extraction. Ponceau S staining (PS) of Rubisco serves as a protein loading control. Representative pictures of the *PcRD18*-transgenic tomato line PcRD18-SlOE and Arabidopsis lines PcRD18-L1/L2/L3 under standard growth conditions are shown in **(D)** and **(E)** respectively.


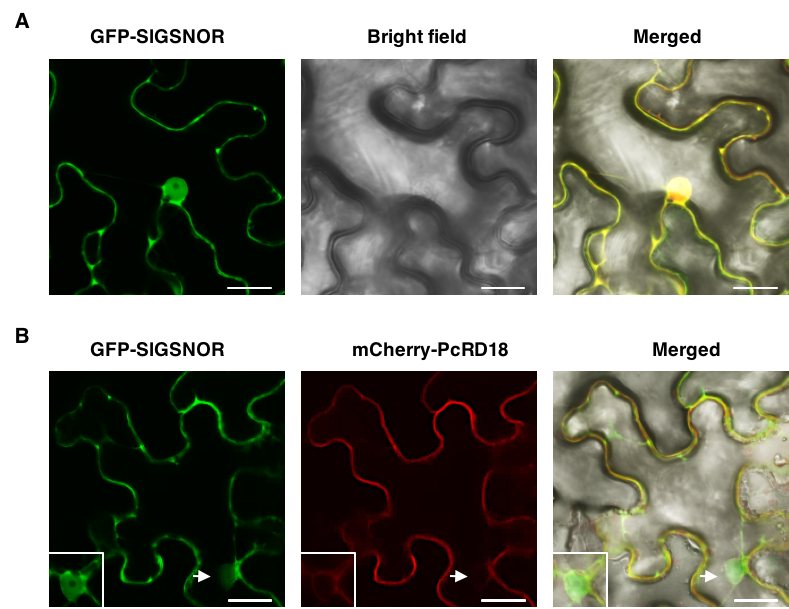


**Figure S3**. Co-localization of GFP-SlGSNOR and mCherry-PcRD18 in *N. benthamiana* leaves. Confocal microscopy images showing the localization of GFP-SlGSNOR **(A)** and co-localization of GFP-SlGSNOR with mCherry-PcRD18 **(B)**. Images were taken at 2 days post agro-infiltration. An enlarged view of the nucleus that is indicated by the white arrow is shown as an insert on the lower left side of the panels in **(B)**. Bars indicate 20 µm. The experiments were carried out at least two times, with similar results.

**
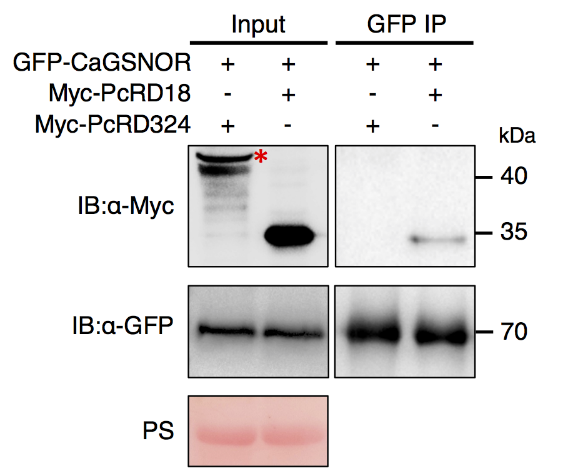
**

**Figure S4**. CaGSNOR-PcRD18 interaction detected by co-IP assay. Total proteins were isolated from *N. benthamiana* leaves 2 days post-agroinfiltration with the indicated constructs. Immunoprecipitation was performed using GFP-Trap beads (GFP-IP). The red asterisk indicates the control effector Myc-PcRD324. Total protein loading normalization is demonstrated by the rubisco band detected via Ponceau staining (PS).


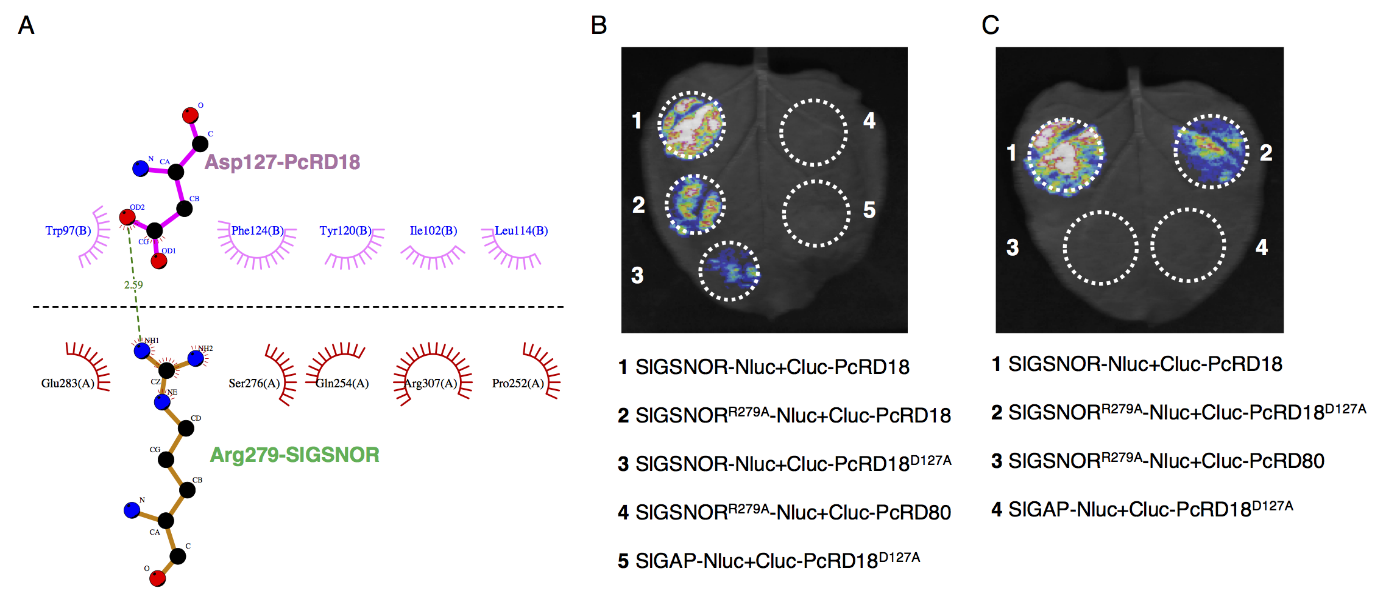


**Figure S5**. Hydrogen bond formation and mutational analysis of the GSNOR/PcRD18 interaction interface. **(A)** The interaction sites in the GSNOR/PcRD18 complex form a hydrogen bond as predicted by LigPlot. The green dashed line indicates the hydrogen bond between Asp127 of PcRD18 and Arg279 of SlGSNOR. **(B-C)** Mutational analysis reveals that alteration of Asp127 in PcRD18 to Ala or Arg279 in SlGSNOR to Ala does not disrupt the interaction between PcRD18 and SlGSNOR, as demonstrated by an LCI assay.


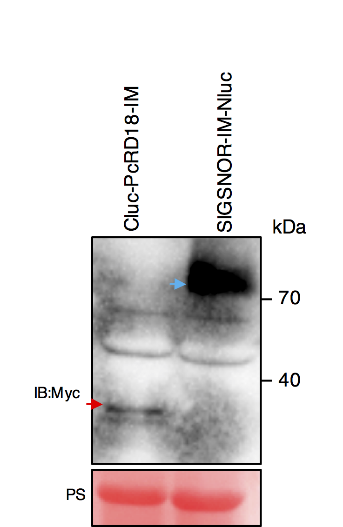


**Figure S6**. Accumulation of Myc-tagged Cluc-PcRD18-IM and SlGSNOR-IM-Nluc proteins upon their transient expression in leaves of *N. benthamiana*. Total proteins were extracted at 2 dpi. The predicted molecular weights for Cluc-PcRD18-IM-Myc (red arrow) and SlGSNOR-IM-Myc-Nluc (blue arrow) are about 33 and 88 kilodaltons (kDa), respectively. Ponceau S staining (PS) of Rubisco serves as a loading control.


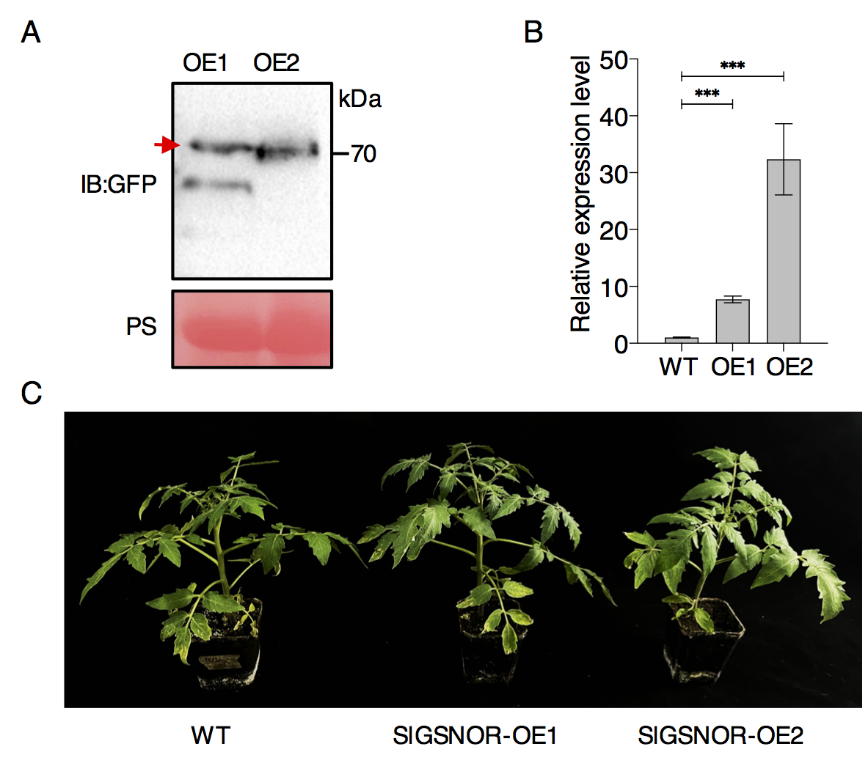


**Figure S7**. Protein detection and morphology of the SlGSNOR overexpressing stable transgenic tomato lines. Detection of GFP-SlGSNOR protein **(A)** and relative expression levels of *SlGSNOR* **(B)** in leaves of the stable SlGSNOR overexpressing tomato lines (SlGSNOR-OE1 and -OE2). Total proteins used for western blotting and RNAs used for RT-qPCR analyses were extracted from leaves of 4-week-old tomato plants. In **(A)** Ponceau S staining (PS) of Rubisco serves as a protein loading control. The predicted molecular weight of GFP-SlGSNOR, indicated with the red arrow, is 70 kilodaltons (kDa). *SlActin* was used as the reference gene and statistical analysis was performed using a two-sided-t-test (n=3; ***, *P* < 0.001) in **(B)**. Error bars indicate the standard deviation. **(C)** Representative picture of 4-week-old SlGSNOR-OE plants. SlGSNOR-OE1 and -OE2 are two independent stable transgenic tomato lines. The experiments were carried out at least two times, with similar results.


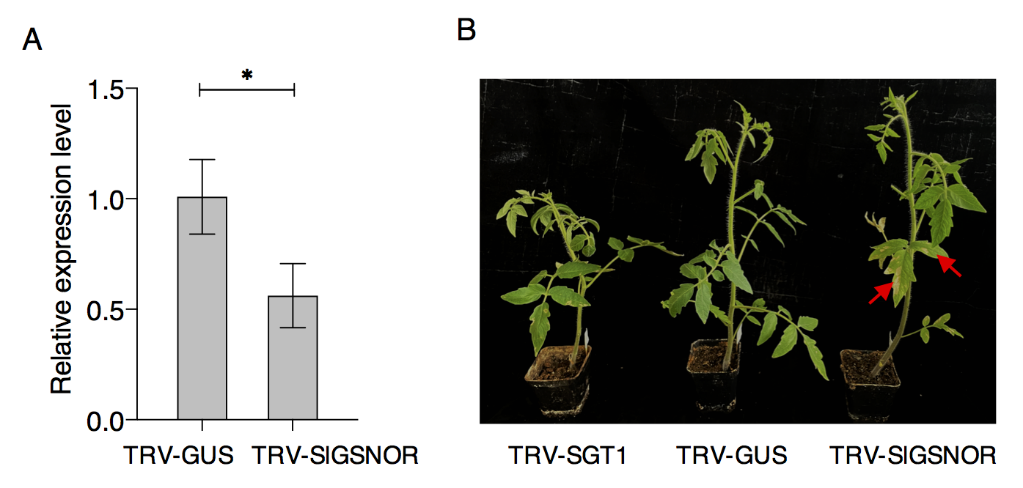


**Figure S8**. Silencing efficiency and morphology of TRV-*SlGSNOR*-inoculated tomato plants. **(A)** The expression levels of *SlGSNOR* in the TRV-*GUS*- (set at 1) and TRV-*SlGSNOR*- inoculated tomato plants were determined by RT-qPCR. Total RNAs were isolated from tomato leaves at 4 weeks post agro-inoculation with agrobacterium carrying the TRV vector. *SlActin* was used for normalization. Statistical analysis was performed using a two-sided-t-test (n=3; *, *P* < 0.05). Error bars indicate the standard deviation. **(B)** Representative picture of the TRV-inoculated plants. The TRV-*SGT1* (Suppressor of G2 allele of Skp1)-inoculated plant, which showed a dwarf phenotype, was used as a control to monitor the silencing process. Red arrows indicate the areas with spontaneous cell death in TRV-*SlGSNOR* leaves. The experiments were carried out at least two times, with similar results.


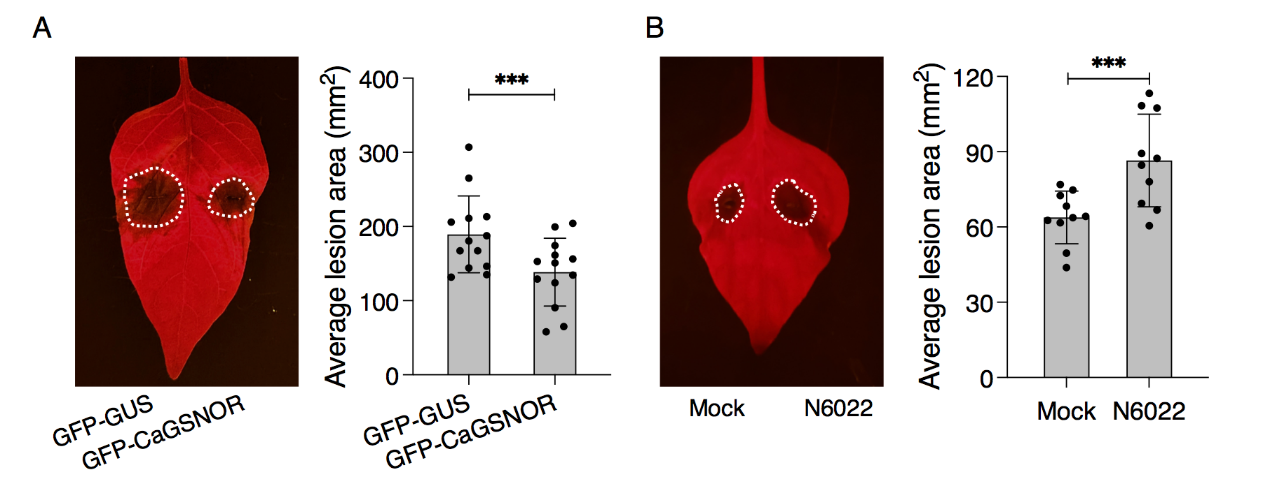


**Figure S9**. CaGSNOR positively regulates pepper resistance to *P. capsici*. **(A)** Transient overexpression of *GFP-CaGSNOR* increases pepper resistance to *P. capsici*. Leaves transiently expressing *GFP-CaGSNOR* or *GFP-GUS* (control) were inoculated with *P. capsici* at 12 hours post agro-infiltration. Lesion areas were quantified at 2 days after inoculation (dai). Representative leaf images (left panel) and statistical analysis (right panel) demonstrate significantly reduced lesions in *GFP-CaGSNOR*-expressing plants (mean ± SD; n=13; paired *t*-test; ***, *P* < 0.001). **(B)** Inhibition of CaGSNOR enzymatic activity compromises pepper resistance to *P. capsici.* Pepper leaves were treated with a 500 μM solution of N6022 (a specific GSNOR inhibitor) or the 0.1% DMSO solvent of the inhibitor, followed by *P. capsici* inoculation 4 hours later. Lesion areas were quantified at 2 dai. Representative leaf images (left panel) show enhanced disease symptoms in N6022-treated leaves. Statistical analysis (right panel) confirmed the presence of significantly enlarged lesion areas in N6022-treated leaves (mean ± SD; n=10; paired *t*-test; ***, *P* < 0.001). White dashed lines indicate the lesion areas.


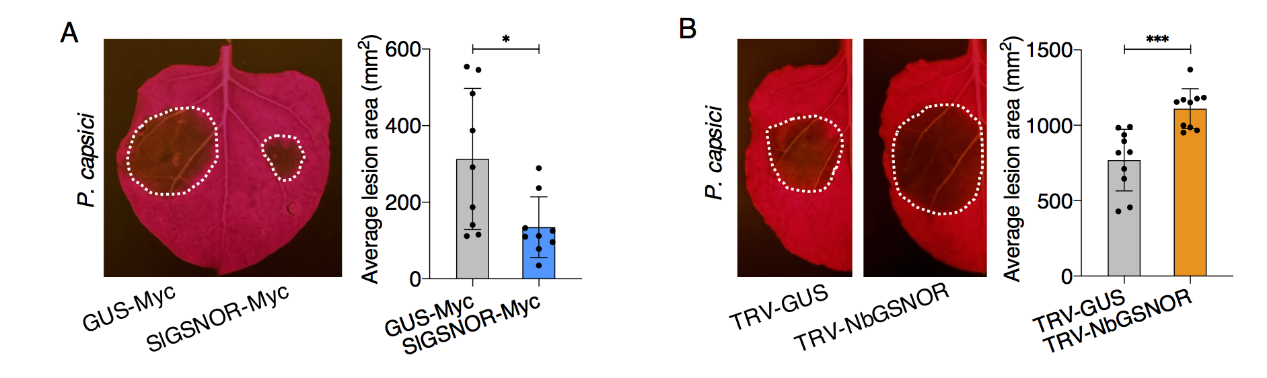


**Figure S10**. Transient overexpression of *SlGSNOR-Myc* and silencing of *NbGSNOR* in *N. benthamiana*. **(A)** Transient overexpression of *SlGSNOR-Myc* significantly decreases *P. capsici* lesion areas, when compared with the *GUS-Myc* control. *N. benthamiana* leaves expressing *SlGSNOR-Myc* and *GUS-Myc* were inoculated with *P. capsici* at 24 hours post agro-infiltration. Lesions were photographed and measured at 2 days after inoculation (dai) (left panel). The bar graph shows the lesion areas analysis (mean ± SD; n=9; paired *t* test; *, *P* < 0.05) (right panel). **(B)** Virus-induced gene silencing (VIGS) of *NbGSNOR* increases *P. capsici* leaf colonization. The middle leaves of TRV-*NbGSNOR*- and TRV-*GUS-*inoculated plants were inoculated with *P. capsici* (left panel). Lesion areas, measured at 2-3 dai, were analyzed and are shown as a bar graph (mean ± SD; n=10; two-sided *t* test; ***, *P* < 0.001) (right panel). White dashed lines indicate the lesion areas.


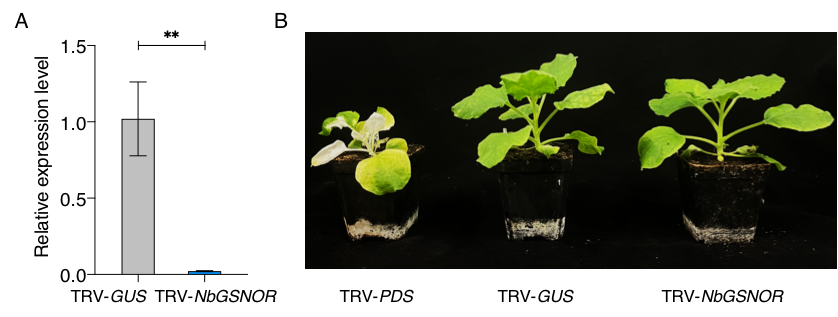


**Figure S11**. Silencing efficiency and morphology of TRV-*NbGSNOR*-inoculated *N. benthamiana* plants. **(A)** The expression level of *NbGSNOR* in TRV-*GUS*- (set at 1) and TRV-*NbGSNOR*-inoculated plants was determined by RT-qPCR. Total RNAs were isolated from *N. benthamiana* leaves at 4 weeks post agro-inoculation with agrobacterium carrying the TRV vector. *NbActin* was used for normalization. Statistical analysis was performed using a two-sided-t-test (n=3; **, *P* < 0.01). Error bars indicate the standard deviation. **(B)** Representative picture of TRV-*GUS*- and TRV-*NbGSNOR*-inoculated *N. benthamiana* plants. The picture was taken at 3 weeks post agro-inoculation with agrobacterium carrying the TRV vectors. TRV-*PDS* (phytoene desaturase)-inoculated plants that develop photobleaching were used to monitor the silencing process. The experiments were carried out at least two times, with similar results.


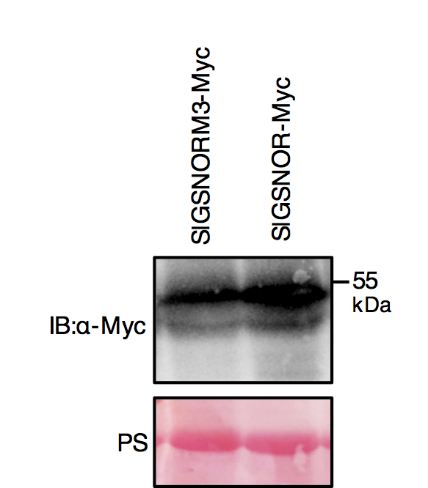


**Figure S12**. Accumulation of the SlGSNOR-Myc and SlGSNORM3-Myc proteins upon transient expression of the encoding genes in leaves of *N. benthamiana*. Total proteins were extracted at 2 days post infiltration. The predicted molecular weights for both SlGSNOR-Myc and SlGSNORM3-Myc are about 55 kilodaltons (kDa). Ponceau S staining (PS) of Rubisco serves as a loading control.


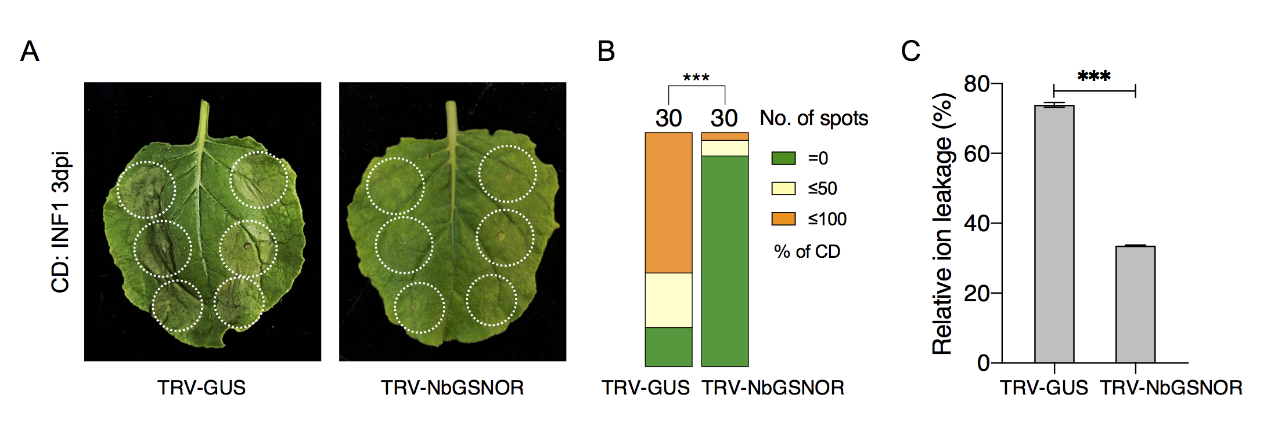


**Figure S13**. Silencing of *NbGSNOR* suppresses INF1-induced plant cell death (CD). INF1 was expressed in the middle, fully expanded leaves of control TRV-*GUS*- or TRV-*NbGSNOR*-inoculated *N. benthamiana* plants. **(A)** Representative photographs were taken at 3 days post infiltration (dpi). White dashed circles indicate the infiltrated areas. **(B)** Analysis of the cell death intensities (one-sided Wilcoxon rank-sum test; ***, *P* < 0.001). **(C)** Relative ion leakage analysis (one-sided *t*-test; ***, *P* < 0.001). Error bars represent the standard deviation. The experiments were carried out at least two times, with similar results.


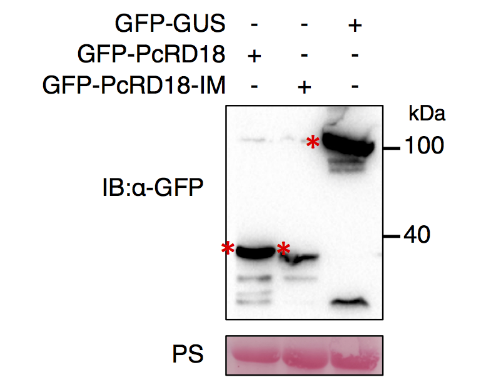


**Figure S14**. Accumulation of the GFP-PcRD18 and GFP-PcRD18-IM proteins upon transient expression of the encoding genes in leaves of *N. benthamiana*. Total proteins were extracted at 2 dpi. Red asterisks mark the intact protein in the corresponding samples. The predicted molecular weights of GFP-GUS, GFP-PcRD18 and GFP-PcRD18-IM are about 100, 40 and 40 kilodaltons (kDa), respectively. Ponceau S staining (PS) of Rubisco serves as a loading control. The experiments were carried out at least two times, with similar results.


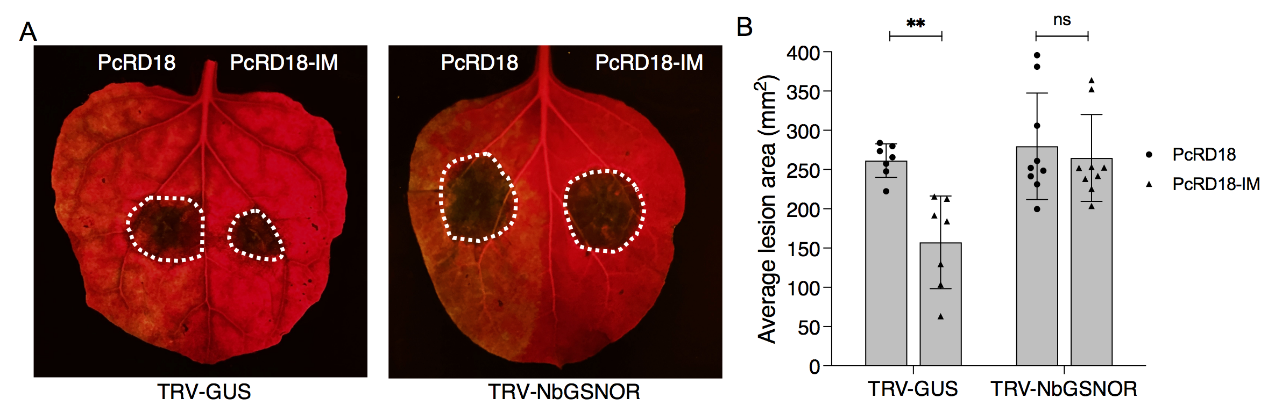


**Figure S15.** PcRD18 requires host GSNOR to enhance susceptibility to *P. capsici*. **(A)** Representative leaves of *N. benthamiana* infected with *P. capsici* following agroinfiltration. Fully expanded leaves of TRV-*GUS* (control) or TRV-*NbGSNOR* (*GSNOR*-silenced) plants were co-infiltrated with *PcRD18* or *PcRD18-IM* on opposite sides (left/right). The leaves were inoculated with *P. capsici* 24 hours post-agroinfiltration. Lesion development was photographed 2 days after inoculation (dai). White dashed lines indicate the lesion areas. **(B)** The bar graph shows the lesion areas analyses. Lesion areas quantified at 2 dai were analyzed by paired *t*-test (mean ± SD; n≥7; **, *P* < 0.01; ns, not significant).


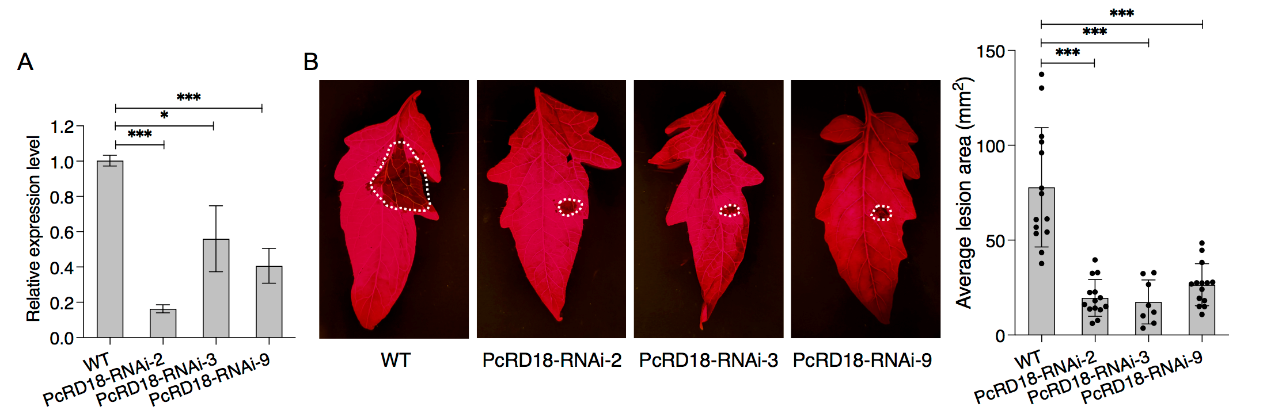


**Figure S16.** Silencing of *PcRD18* in *P. capsici* diminishes its virulence on tomato. **(A)** Relative expression levels of *PcRD18* in three independent transformed strains (PcRD18-RNAi-2 -3 and -9) and the wild type (*P. capsici* BYA5 strain). Total RNA was extracted from the mycelium of *P. capsici* and RT-qPCR analysis was employed to quantify the transcript levels of *PcRD18* in each sample, with *PcActin* serving as the normalization control. Statistical analysis was conducted using *t*-tests (mean ± SD; n=3; *, *P* < 0.05; ***, *P* < 0.001). **(B)** Stable silencing of *PcRD18* in *P. capsici* leads to a decreased virulence on tomato. Detached leaves were inoculated with *P. capsici* and photographed at 2 days after inoculation. Bar graphs depict the average lesion areas, with statistical analysis conducted using *t*-tests (mean ± SD; n≥8; ***, *P* < 0.001). White dashed lines delineate the lesion areas.

**Figure S17.** Transient expression of *SlGSNOR-IM* in leaves of *NbGSNOR*-silenced *N. benthamiana* decreases lesion formation by *P. capsici* when compared to wild-type *SlGSNOR*. *GFP-SlGSNOR-IM* and *GFP-SlGSNOR* were expressed at the indicated sites of TRV-*NbGSNOR*-inoculated leaves, followed by inoculation with *P. capsici* 12 hours later. Lesions were then photographed and their area was measured 2 days post-inoculation. Statistical analysis of the average lesion areas was performed with paired *t*-tests (mean ± SD; n≥9; ***, *P* < 0.001). White dashed lines indicate the lesion areas.

**
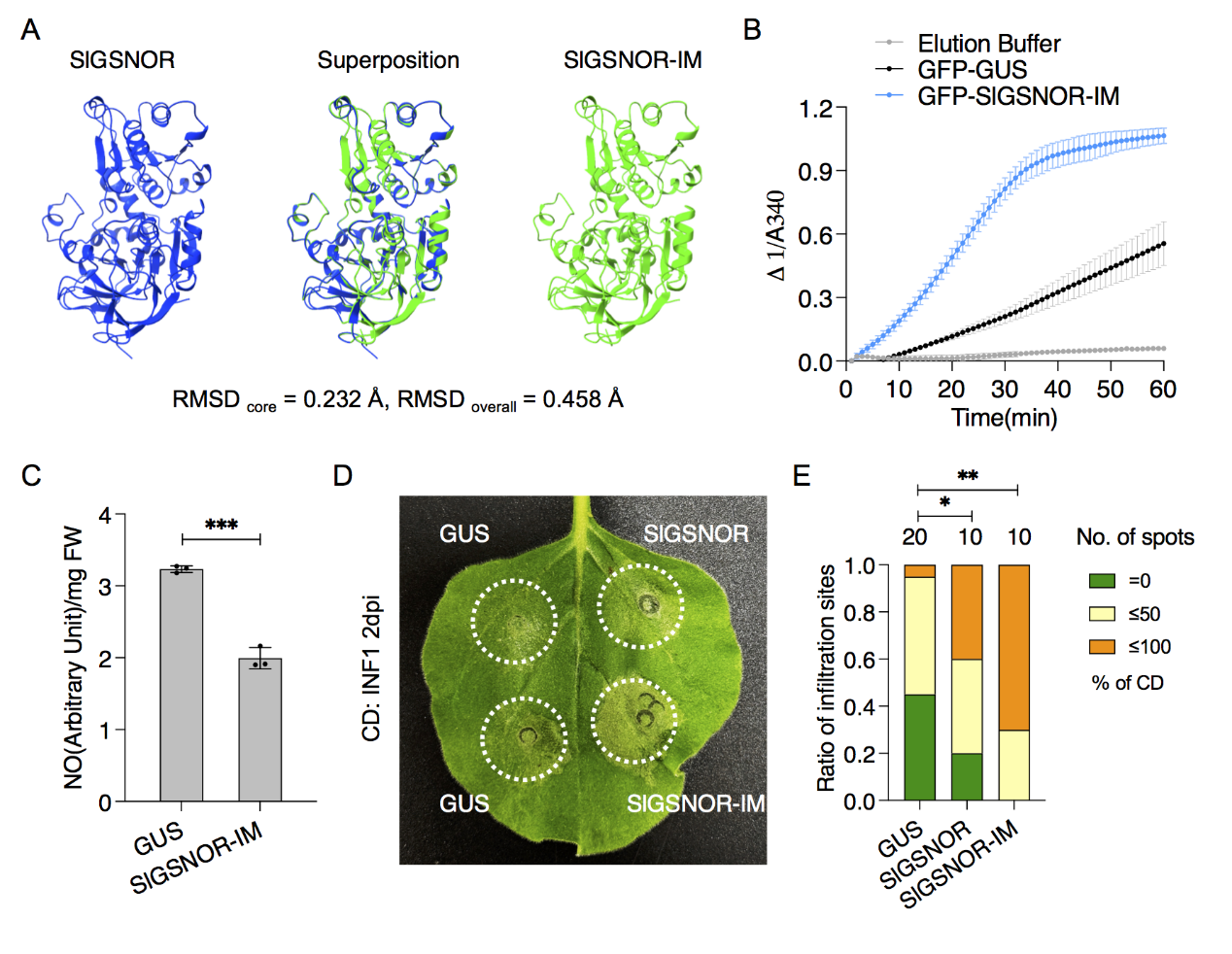
**

**Figure S18.** Structure prediction and functional validation of SlGSNOR-IM. **(A)** Comparison of the predicted three-dimensional structures of SlGSNOR and SlGSNOR-IM. RMSD, root mean square deviation of the Cα atomic coordinates of superimposed molecules. **(B)** SlGSNOR-IM overexpression enhances *S*-nitrosoglutathione (GSNO) reductase activity when compared to the mock control. Proteins were extracted from *N. benthamiana* leaves expressing GFP-SlGSNOR-IM or GFP-GUS at 2 days post ago-infiltration (dpi). Elution buffer is used as the negative control. The relative enzyme activity was determined by NADH consumption, as indicated by △1/A340. Data are presented as mean ± SD (n=3). **(C)** SlGSNOR-IM overexpression decreases cellular NO levels. GFP-SlGSNOR-IM and GFP-GUS were transiently expressed in *N. benthamiana* leaves after which NO levels were measured using the NO-sensitive probe DAF-2 DA, at 2 dpi. Statistical analysis was conducted using *t*-tests (mean ± SD; ***, *P* < 0.001). **(D)** SlGSNOR-IM enhances INF1-triggered immunity. *GFP-SlGSNOR-IM*, *GFP-SlGSNOR* and *GFP-GUS* were co-expressed with *INF1* in *N. benthamiana.* Representative photographs were taken at 2 days post infiltration (dpi). **(E)** Analysis of the cell death intensities. The plant cell death (CD) levels were divided over three categories, according to the percentage of necrotic area that developed in the infiltrated area (0%, 0–50%, and 50–100% cell death). Statistical analysis was conducted using a Kruskal-Wallis test (*, *P* < 0.05; **, *P* < 0.01).

**Figure S19.** Conservation of sites targeted by PcRD18 on SlGSNOR, across different plant species. An alignment of GSNOR sequences from *Solanum lycopersicum*, *S. tuberosum*, *Nicotiana benthamiana*, *Arabidopsis thaliana*, *Spinacia oleracea*, *Oryza sativa*, *Zea mays*, *Brachypodium distachyon*, *Ananas comosus*, *Nymphaea colorata*, and *Physcomitrella patens* was performed using Muscle 5.1. The red arrowheads indicate the amino acids of SlGSNOR targeted by PcRD18. Black shading is used to indicate identical amino acids, whereas grey shading denotes similar amino acids.

**Table S1.** Identification of PcRD18-interacting proteins by protein immunoprecipitation, followed by tryptic digestion and analysis of the peptides by mass spectrometry.

**Table S2.** DNA sequence of the primers used in this study.
